# Synergistic experimental and theoretical investigation of bis-chalcone derivatives as efficient corrosion inhibitors for carbon steel in acidic media

**DOI:** 10.1038/s41598-026-60964-2

**Published:** 2026-07-18

**Authors:** Basma Hussein, Mohammed M. El-Gamil, Ahmed A. Noser, Marwa N. El-Nahass

**Affiliations:** 1 Department of Chemistry, Faculty of Science, Tanta University, Tanta, 31527 Egypt; 2 Forensic Medicine, Mansoura Laboratory, Department of Toxic and Narcotic Drug, Medico Legal Organization, Ministry of Justice, Mansoura, Egypt

**Keywords:** Corrosion inhibition, Carbon steel, Bis-chalcone dyes, Physisorption, Potentiodynamic polarization, Electrochemical impedance spectroscopy, Thermodynamic parameters, Chemistry, Materials science

## Abstract

Building on our previous investigations of chalcone-based functional materials, the present work reports a synergistic experimental and theoretical investigation of the designed bis-chalcone derivatives, namely 3-(4-(dimethylamino)benzylidene)pentane-2,4-dione (DBPD) and 3-(3-(4-dimethylamino)phenylallylidene)pentane-2,4-dione (DPAPD), as highly efficient corrosion inhibitors for carbon steel in 1.0 M HCl solution. The inhibition performance and adsorption behavior were evaluated using weight loss, potentiodynamic polarization, electrochemical impedance spectroscopy, surface characterization techniques (SEM/EDX), density functional theory, and Monte Carlo simulations. Both compounds exhibited excellent concentration-dependent corrosion inhibition efficiency. Potentiodynamic polarization measurements confirmed that both compounds behave as mixed-type corrosion inhibitors, significantly reducing the corrosion current density (I_corr_) from 217.59 to 27.61 and 21.18 μA cm⁻², while electrochemical impedance spectroscopy (EIS) revealed a marked increase in the charge-transfer resistance (R_ct_) from 19.7 to 219.05 and 244.78 Ω cm² in the presence of 10 × 10⁻⁵ M DBPD and DPAPD, respectively, confirming the formation of a protective adsorbed film on the steel surface. Adsorption followed the Langmuir isotherm model (R^2^ > 0.994), indicating spontaneous adsorption and nearly homogeneous surface coverage. SEM and EDX analyses confirmed the formation of a compact protective film and a substantial reduction in corrosion damage. DFT calculations and MC simulations revealed strong adsorption of the inhibitor molecules on the Fe(110) surface, with DPAPD exhibiting higher adsorption affinity, in excellent agreement with experimental observations. The enhanced inhibition performance was attributed to the electron-donating dimethylamino group, extended π-conjugation, and multiple adsorption centers. These findings provide molecular-level insight into the inhibition mechanism and highlight bis-chalcone derivatives as promising, structurally tunable corrosion inhibitors for acidic industrial applications.

**Supplementary Information:**

The online version contains supplementary material available at 10.1038/s41598-026-60964-2.

## Introduction

Carbon steel is one of the most widely used engineering materials owing to its favorable mechanical properties, low cost, and ease of fabrication. Consequently, it has found extensive applications in pipelines, storage tanks, heat exchangers, petrochemical facilities, and numerous industrial infrastructures^1–3^. Despite these advantages, carbon steel remains highly susceptible to corrosion in acidic environments, particularly during industrial operations such as acid pickling, descaling, and cleaning processes employing hydrochloric acid solutions^4^. Corrosion-related degradation not only results in severe economic losses associated with maintenance and equipment replacement, but also poses serious environmental and safety concerns due to possible structural failure of industrial systems and transportation networks^5^.

Carbon steel was selected as the investigated substrate because of its extensive industrial utilization and its high vulnerability to corrosion in acidic media. A 1.0 M HCl solution was employed as the aggressive corrosive medium since hydrochloric acid is widely used in acid treatment and cleaning processes and is considered a standard medium for evaluating corrosion inhibition performance under severe acidic conditions. Moreover, the use of 1.0 M HCl facilitates reliable comparison with previously reported corrosion inhibition studies available in the literature.

Among the various corrosion protection strategies, the use of corrosion inhibitors is considered one of the most practical and economically effective methods for minimizing metallic degradation in aggressive media^6–8^. Corrosion inhibitors function mainly through adsorption onto the metallic surface, forming a protective barrier that suppresses both anodic metal dissolution and cathodic reduction reactions. In recent years, organic corrosion inhibitors have attracted considerable interest because of their high inhibition efficiency, structural tunability, and strong adsorption capability^9–11^. Their inhibition performance is generally associated with the presence of heteroatoms such as nitrogen, oxygen, and sulfur, in addition to π-electron systems and conjugated aromatic structures that facilitate electron donation and strong interaction with the metal surface^12–14^.

Among the different classes of organic compounds, chalcone derivatives have emerged as highly promising corrosion inhibitors for metallic materials in acidic environments^15–17^. Chalcones are characterized by an α,β-unsaturated carbonyl framework connecting two aromatic rings through an extended π-conjugated system, which enhances electron delocalization and adsorption affinity toward metal surfaces. Furthermore, the incorporation of electron-donating substituents and heteroatom-containing functional groups within the chalcone skeleton significantly improves the adsorption strength through donor–acceptor interactions with iron atoms^18,19^. The planar molecular geometry and conjugated structure also facilitate the formation of compact and adherent protective films that effectively hinder the penetration of corrosive species to the metal surface.

Several investigations have demonstrated that chalcone-based compounds exhibit excellent corrosion inhibition efficiencies for carbon steel in acidic media, often at relatively low inhibitor concentrations^20–24^. The inhibition mechanism is generally attributed to adsorption processes involving combined physical and chemical interactions that suppress charge-transfer reactions and block active corrosion sites^25^. Moreover, the presence of hydrophobic substituents and extended π-conjugation can significantly enhance surface coverage and improve the stability of the adsorbed protective layer^26^. Nevertheless, most previously reported chalcone inhibitors were mainly evaluated through conventional electrochemical measurements without establishing a comprehensive correlation between molecular structure, electronic properties, adsorption behavior, and inhibition performance at the molecular level.

Recent studies have demonstrated the importance of combining experimental and theoretical approaches, including density functional theory (DFT), molecular dynamics (MD), and Monte Carlo (MC) simulations, to elucidate corrosion inhibition mechanisms at the molecular level. Electronic structure calculations and molecular simulations have proven highly effective in correlating quantum chemical descriptors with adsorption behavior and inhibition performance, thereby providing deeper understanding of inhibitor–metal interactions and protective film formation processes^27,28^. Such integrated approaches enable not only validation of experimental observations but also the prediction of structure–activity relationships that can guide the rational design of next-generation corrosion inhibitors.

Despite the remarkable progress achieved in the development of organic corrosion inhibitors, systematic investigations on bis-chalcone derivatives integrating advanced electrochemical techniques, surface characterization, and theoretical simulations remain scarce^29–31^. In particular, the influence of molecular conjugation length, electron-donating substituents, and adsorption-active centers on adsorption affinity and corrosion inhibition performance has not been sufficiently clarified. Furthermore, only limited studies have combined experimental corrosion analysis with density functional theory and Monte Carlo simulations to establish predictive structure–performance relationships for chalcone-based inhibitors. Consequently, significant challenges remain in understanding how subtle molecular structural modifications influence adsorption behavior and protective film formation at the metal/solution interface.

Beyond the development of efficient corrosion inhibitors, there is an increasing need for studies that provide fundamental molecular-level insight into corrosion inhibition mechanisms. Establishing direct relationships between molecular structure, electronic properties, adsorption characteristics, and corrosion performance is essential for advancing corrosion science from empirical inhibitor screening toward predictive inhibitor design. Therefore, integrated experimental–computational investigations are of particular importance because they not only improve inhibitor performance but also contribute to a deeper mechanistic understanding of adsorption phenomena and interfacial processes occurring on metallic surfaces.

In continuation of our previous work on chalcone-based functional materials^32^, the present study reports a comprehensive experimental and theoretical investigation of two synthesized bis-chalcone derivatives, namely 3-(4-(dimethylamino)benzylidene)pentane-2,4-dione (DBPD) and 3-(3-(4-dimethylamino)phenylallylidene)pentane-2,4-dione (DPAPD), as corrosion inhibitors for carbon steel in 1.0 M HCl solution. Furthermore, the effect of temperature on the corrosion behavior was systematically investigated to gain deeper insight into the thermodynamic and kinetic aspects governing the adsorption process. The temperature range of 298–318 K was selected to evaluate the influence of temperature on adsorption behavior, inhibition efficiency, and thermodynamic activation parameters under conditions relevant to industrial applications.

The novelty of this work lies in establishing direct correlations between molecular structure, electronic characteristics, adsorption behavior, and corrosion inhibition efficiency through the combined application of weight loss measurements, potentiodynamic polarization, electrochemical impedance spectroscopy, SEM/EDX surface characterization, density functional theory calculations, and Monte Carlo simulations. Particular emphasis was devoted to elucidating the influence of electron-donating dimethylamino substituents, extended π-conjugation, and multiple adsorption-active centers on inhibitor–metal interactions and protective film formation on the Fe(110) surface. Unlike many previously reported chalcone-based corrosion inhibitors, the present work provides a comprehensive mechanistic interpretation supported by both experimental observations and theoretical calculations. The combined findings deliver new molecular-level insight into the adsorption mechanism of bis-chalcone derivatives, establish predictive structure–performance relationships, and contribute to the rational design of highly efficient and structurally tunable corrosion inhibitors for acidic industrial environments.

## Experimental

### Materials

All chemicals used in the present study were of analytical grade and obtained from commercial suppliers; they were used as received without further purification. A 30% hydrochloric acid solution was employed to prepare the corrosive medium.

Carbon steel specimens were prepared from sheet material, and a set of seven samples with identical dimensions was obtained for experimental consistency. The chemical composition of the steel (wt%) was determined as follows: C (0.076), Mn (0.19), P (0.012), Si (0.026), Cr (0.05), and Al (0.023), with iron representing the balance.

Prior to each experiment, the steel surfaces, including those used as working electrodes, were carefully prepared through mechanical polishing using emery papers of progressively increasing fineness (400–1200 grit) until a smooth and reflective surface was achieved. The polished specimens were then rinsed thoroughly with deionized water to eliminate any adhered particles or residues.

All test solutions were freshly prepared using distilled water to ensure reproducibility. Stock solutions of hydrochloric acid and the investigated bis-chalcone inhibitors were initially prepared and subsequently diluted to the desired concentrations for each experiment. Corrosion measurements were carried out in naturally aerated acidic solutions under static conditions (without stirring), containing different concentrations of the synthesized inhibitors.

### Solutions

The corrosive medium used throughout this study was a 1.0 M hydrochloric acid solution, prepared by dilution of concentrated HCl (30%) with distilled water. Corrosion measurements were carried out in this acidic environment in both the absence and presence of different concentrations of the synthesized bis-chalcone inhibitors, DBPD and DPAPD, within the concentration range of 1 × 10^−6^ to 10 × 10⁻^5^ M.

Stock solutions of the investigated inhibitors (1 × 10⁻^3^ M) were initially prepared using ethanol as a solvent to ensure complete dissolution, and subsequently diluted to the required concentrations for experimental use. All test solutions were freshly prepared immediately before each measurement to maintain reliability and consistency of the results.

### Weight-loss measurements

The corrosion inhibition performance of the investigated compounds was evaluated using the weight loss (gravimetric) method according to standard procedures^33^. The corrosive medium consisted of a 1.0 M hydrochloric acid solution prepared by dilution of concentrated HCl (30%) with distilled water.

Carbon steel specimens with dimensions of 2 × 3 × 0.2 cm were mechanically polished, thoroughly cleaned, and accurately weighed prior to immersion. The prepared coupons were then immersed in 50 mL of the test solution in both the absence and presence of different concentrations (1 × 10^−6^ to 10 × 10⁻^5^ M) of the inhibitors DBPD and DPAPD. The exposure times were varied at 30, 60, 90, 120, and 180 min under naturally aerated conditions.

After each immersion interval, the specimens were removed, rinsed with distilled water to eliminate any residual solution, dried under ambient conditions, and reweighed. All measurements were performed in duplicate to ensure reproducibility, and the reported values represent the average of the obtained results.

To examine the influence of temperature on the corrosion process, additional experiments were conducted over the temperature range of 298–318 K using a thermostatically controlled water bath, while maintaining a fixed immersion period of 3 h.

The weight loss (W) was determined from the difference between the initial and final mass of the specimens according to the following relation^3^:

1 $${\mathrm{W}} = \left( {{\mathrm{m}}_{{2}} {-}{\text{ m}}_{{1}} } \right)$$

where m_1_ and m_2_ are the weights of before and after exposure to the corrosive solution, respectively. The corrosion rate (CR), inhibition efficiency (IE %) and surface coverage (θ) were determined according to Eqs. (2–4):

2 $${\mathrm{CR}} = {\mathrm{W}}/{\mathrm{A}} \times {\mathrm{t}}$$

3 $${\mathrm{IE}}\% { = }\left( {{\mathrm{w}}_{{\mathrm{o}}} - {\mathrm{w}}_{{\mathrm{i}}} {\text{/ w}}_{{\mathrm{o}}} } \right) \, \times {100}$$

4 $$\theta = {1} - {\mathrm{w}}_{{\mathrm{i}}} /{\mathrm{w}}_{{\mathrm{o}}}$$

where *W* represents the weight loss of carbon steel (mg), *A* is the surface area of the specimen (cm^2^), and *t* denotes the immersion time (min). *W₀* and *Wᵢ* correspond to the weight loss of the carbon steel specimen in the absence and presence of different inhibitor concentrations, respectively.

### Electrochemical measurements

Electrochemical measurements were performed using a CS350 electrochemical workstation in a conventional three-electrode configuration. A platinum electrode was used as the counter electrode, while a saturated Ag/AgCl electrode served as the reference electrode. The working electrode was fabricated from carbon steel with a defined exposed surface area of 1.0 cm^2^.

Prior to each experiment, the working electrode surface was mechanically polished using emery papers of progressively finer grades, followed by ultrasonic cleaning in ethanol to remove surface contaminants, and finally rinsed with distilled water. All reported potentials in this study are referenced to the Ag/AgCl electrode.

The electrochemical measurements were carried out at 25 °C in naturally aerated 1.0 M HCl solutions, in both the absence and presence of different concentrations of the investigated inhibitors. Prior to data acquisition, the working electrode was immersed in the test solution for 30 min to allow stabilization of the open-circuit potential (OCP). Measurements were initiated only after the OCP drift became negligible, indicating the attainment of a quasi-steady state and establishment of adsorption equilibrium of inhibitor molecules on the metal surface.

Potentiodynamic polarization measurements were conducted at a scan rate of 1 mV s⁻^1^ over a potential range from − 0.6 to 0.2 V with respect to the OCP. The corrosion parameters were extracted from the polarization curves, and the inhibition efficiency as well as the surface coverage were calculated using the following relations^34^:

5 $$\% {\mathrm{IE}} = \theta \times {1}00 = \left( {{1} - \left( {{\mathrm{i}}_{{{\mathrm{corr}}({\mathrm{inh}})}} /{\mathrm{i}}_{{{\mathrm{corr}}({\mathrm{free}})}} } \right)} \right) \times {1}00$$

where, i_corr_ and i_corr(inh)_ represent the corrosion current densities in the absence and presence of inhibitor, respectively. These values were obtained by extrapolation of anodic and cathodic Tafel regions. Electrochemical impedance spectroscopy (EIS) measurements were performed over a frequency range of 1 Hz to 100 kHz using a sinusoidal perturbation of 10 mV amplitude under the same experimental conditions. The frequency range was selected to fully capture all electrochemical relaxation processes at the metal/solution interface, ensuring accurate separation of solution resistance, charge transfer resistance, and double-layer capacitance contributions. The high-frequency response accounts for ohmic resistance, whereas the low-frequency limit allows complete development of interfacial polarization and diffusion-related processes, which are essential for reliable fitting using equivalent circuit models.

The inhibition efficiency^34^ was calculated from the charge transfer resistance values according to:

6 $$\% {\mathrm{IE}} = \left( {{1} - \left( {{\mathrm{R}}_{{{\mathrm{ct}}}} /{\mathrm{R}}_{{{\mathrm{ct}}({\mathrm{inh}})}} } \right)} \right) \times {1}00$$

where Rct and R_ct(inh)_ in absence and presence of inhibitor, respectively.

### Surface examinations

The surface characteristics of carbon steel samples after 24 h exposure to 1.0 M HCl solution were analyzed using a JEOL scanning electron microscope (SEM, JSM-6390) equipped with an energy-dispersive X-ray (EDX) detector. The examination was performed for specimens immersed in the acidic medium both without inhibitor and in the presence of higher concentrations of the studied dye compounds.

### Computational study

The adsorption process of an inhibitor as neutral compounds as well as protonated compounds [DBPD-N^+^(CH_3_)_2_ and DPAPD-N^+^(CH_3_)_2_] on an iron surface is examined using Monte Carlo simulations using the adsorption locator module from Accelrys Inc.^35^. We chose Fe(110) to replicate the adsorption process because the three kinds of Fe surfaces (110, 100, 111), Fe(111) and Fe(100), have comparatively open structures, but Fe(110) is a density packed surface with the highest stability^36,37^. It was created by cleaving a bcc Fe crystal, then enlarging it to a (8 × 8) supercell, and lastly building a 25 Å thickness vacuum slab above the Fe(110) plane. The interaction between the Fe (110) surface and the investigated inhibitors monomer molecules (1–8 #inhibitors monomer) is carried out in a simulation box of 19.809 Å × 19.809 Å × 31.065 Å to elucidate the effect of inhibitor concentration. To obtain an equilibrium configuration of the inhibitor/Fe(110) system, the optimized inhibitor molecules were allowed to adsorb on the refined Fe(110) surface utilizing a simulated annealing job. Finally, the adsorption energy for the most stable inhibitor/Fe(110) system configuration may be calculated. To simulate solvent effects, the application of COMPASS-III (Condensed-phase Optimized Molecular Potentials for Atomistic Simulation Studies) to optimize the constructions of inhibitor in addition, 350 H_2_O, 5H_3_O^+^ and 5HCl molecules which were included in adsorption system constitutes a technical advance in the forcefield approach^38^. It is the first ab-initio forcefield to predict chemical characteristics (structural, conformational, vibrational, etc.) as well as condensed-phase properties (equation of state, cohesive energies, etc.) for a wide range of chemical systems.

## Results and discussion

### Weight-loss measurements

**Fig. 1:**
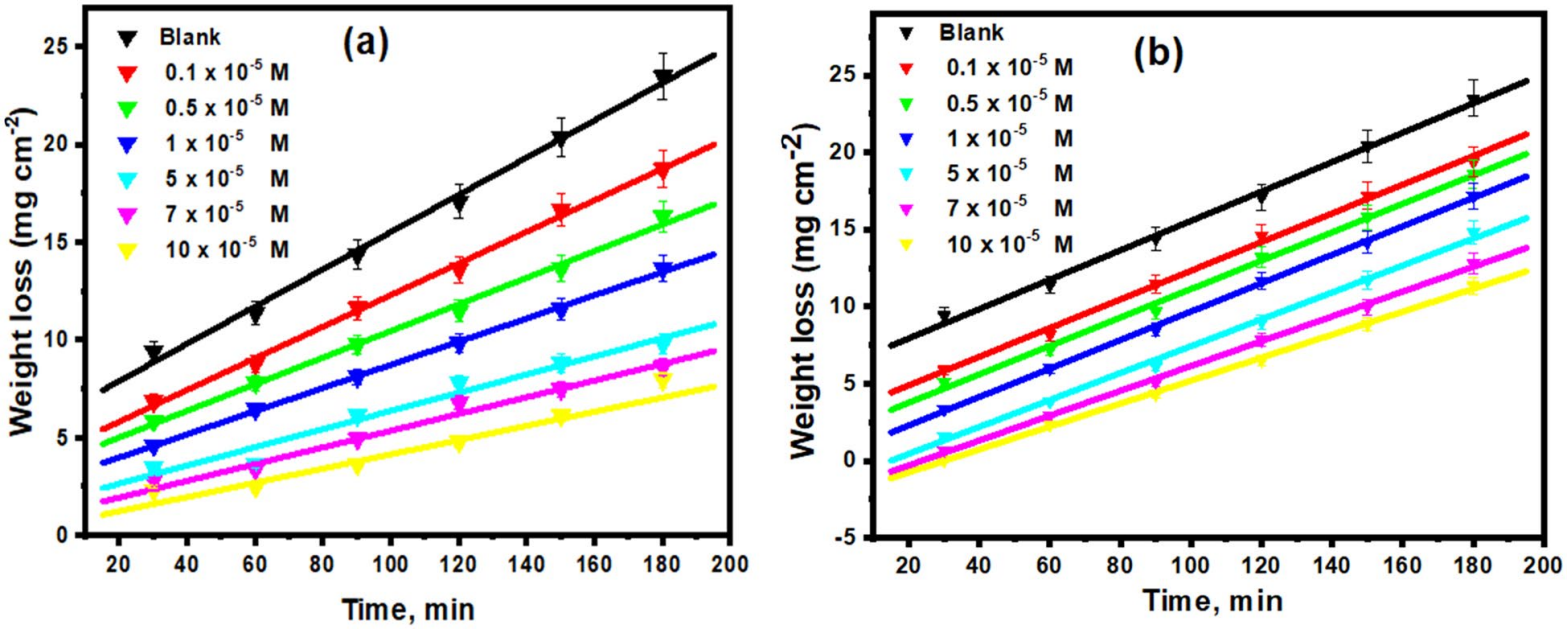
Weight-loss—time curves for the dissolution of carbon steel in 1.0 M HCl in the absence and presence of the inhibitors (**a**) DBPD and (**b**) DPAPD at different concentrations (0.1 × 10^–5^ to 10 × 10^–5^ M) at 25 °C.

Figure 1 presents the variation of weight loss of carbon steel as a function of immersion time in 1.0 M HCl solution at 25 °C, in both the absence and presence of different concentrations of the investigated bis-chalcone inhibitors, DBPD and DPAPD. As observed, the mass loss increases steadily with increasing exposure time for all tested systems, exhibiting an approximately linear relationship. This behavior indicates a nearly constant corrosion rate over the examined time interval.

The introduction of the inhibitors leads to a marked reduction in weight loss compared to the uninhibited solution, confirming their ability to effectively suppress the corrosion of carbon steel in acidic medium. Moreover, the inhibition effect becomes increasingly significant with rising inhibitor concentration, reflecting enhanced protective performance.

The linear dependence of weight loss on immersion time suggests that no substantial corrosion product film is formed on the metal surface during the exposure period. Instead, the observed inhibition behavior can be attributed mainly to the adsorption of inhibitor molecules onto the steel surface. The adsorbed layer acts as a protective barrier, limiting the direct interaction between the metal surface and the aggressive acidic environment, thereby reducing the corrosion rate. Increasing the inhibitor concentration (1 × 10^−6^–10 × 10⁻^5^ M) enhances surface coverage, which in turn improves the inhibition efficiency^39^.

The inhibition efficiency values obtained at different concentrations and temperatures (25–45 °C) are summarized in Tables 1 and 2. It is evident that the efficiency increases with increasing inhibitor concentration, which can be ascribed to the greater availability of inhibitor molecules for adsorption. In contrast, a decrease in inhibition efficiency is observed with increasing temperature, which may be attributed to partial desorption of the adsorbed molecules and a consequent weakening of the inhibitor–metal interaction at elevated temperatures.

**Table1:** Data of weight-loss measurements for carbon steel in 1.0 M HCl solution in the absence and presence of different concentrations of investigated inhibitors at 25 °C.

Inhibitor	DBPD					DPAPD				
Conc (M)	Weight loss (mg cm^-2^)	Mean ± SD	CR (mg cm^-2^ min^-1^)	Ɵ	%IE	Weight loss (mg cm^-2^)	Mean ± SD	CR (mg cm^-2^ min^-1^)	Ɵ	%IE
Blank (1.0 M HCl )	23.5	16.03 ± 5.36	0.130	–	–	23.5	16.03 ± 5.36	0.130	–	–
0.1 × 10^–5^	18.76	12.73 ± 4.54	0.104	0.705	70.5	19.4	12.79 ± 5.22	0.107	0.807	80.7
0.5 × 10^–5^	16.33	10.83 ± 3.82	0.090	0.726	72.6	18.6	11.60 ± 5.18	0.103	0.824	82.4
1 × 10^–5^	13.66	9.06 ± 3.32	0.075	0.741	74.1	17.16	10.13 ± 5.17	0.095	0.837	83.7
5 × 10^–5^	9.83	6.63 ± 2.65	0.054	0.835	83.5	14.8	7.83 ± 4.95	0.082	0.877	87.7
7 × 10^–5^	8.66	5.70 ± 2.32	0.048	0.865	86.5	12.8	6.55 ± 4.52	0.071	0.897	89.7
10 × 10^–5^	8.00	4.57 ± 2.21	0.044	0.889	88.9	11.33	5.57 ± 4.18	0.062	0.912	91.2

**Table 2:** Data of weight-loss measurements for carbon steel in 1.0 M HCl solution in the absence and presence of different concentrations of investigated inhibitors at different temperatures.

		30 °C		35 °C		40 °C		45 °C	
Inhibitor	Conc × 10^–5^ (M)	Ɵ	%IE	Ɵ	%IE	Ɵ	%IE	Ɵ	%IE
DBPD	0.1	0.691	69.1	0.671	67.1	0.628	62.8	0.614	61.4
	0.5	0.707	70.7	0.679	67.9	0.659	65.9	0.643	64.3
	1	0.718	71.8	0.706	70.6	0.687	68.7	0.665	66.5
	5	0.821	82.1	0.816	81.6	0.806	80.6	0.793	79.3
	7	0.852	85.2	0.842	84.2	0.838	83.8	0.829	82.9
	10	0.881	88.1	0.873	87.3	0866	86.6	0.861	86.1
DPAPD	0.1	0.801	80.1	0.793	79.3	0.784	78.4	0.773	77.3
	0.5	0.811	81.1	0.804	80.4	0.794	79.4	0.785	78.5
	1	0.823	82.3	0.813	81.3	0.806	80.6	0.797	79.7
	5	0.871	87.1	0.864	86.4	0.858	85.8	0.85	85.0
	7	0.891	89.1	0.882	88.2	0.876	87.6	0.869	86.9
	10	0.908	90.8	0.902	90.2	0.897	89.7	0.89	89.0

A comparison between the two studied compounds reveals that DPAPD consistently exhibits higher inhibition efficiency than DBPD at equivalent concentrations, particularly at 10 × 10⁻^5^ M. This superior performance can be associated with its stronger adsorption affinity and the formation of a more stable and protective adsorbed layer on the carbon steel surface.

### Adsorption isotherms

Adsorption isotherms provide valuable insight into the corrosion inhibition mechanism, as they offer a quantitative description of the interaction between inhibitor molecules and the metal surface. The adsorption of organic inhibitors on carbon steel generally occurs through two main pathways: physisorption, which is driven by electrostatic interactions, and chemisorption, which involves charge sharing or electron transfer between the inhibitor molecules and the metal surface. The dominant adsorption mode is influenced by several factors, including the molecular structure of the inhibitor, the characteristics of the corrosive medium, and the physicochemical properties of the metal surface, particularly its charge distribution.

The degree of surface coverage (θ) of carbon steel by the inhibitor molecules was calculated from weight loss data according to Eq. (5). To gain deeper insight into the adsorption behavior at 25 °C, the obtained θ values were analyzed using several adsorption isotherm models, including Langmuir, Temkin, Frumkin, and Flory–Huggins isotherms. Among these models, the experimental data exhibited the best linear correlation with the Langmuir isotherm, suggesting that the adsorption process proceeds via the formation of a uniform monolayer on the metal surface, with negligible lateral interaction between the adsorbed species^40^.

Accordingly, the adsorption behavior of the studied inhibitors can be described using the Langmuir isotherm equation^40^;

7 $$\theta /\left( {{1} - \theta } \right) = {\mathrm{K}}_{{{\mathrm{ads}}}} \times {\text{ C}}$$

where C is the inhibitor concentration and K_ads_ the equilibrium constant of the adsorption process.

**Fig. 2:**
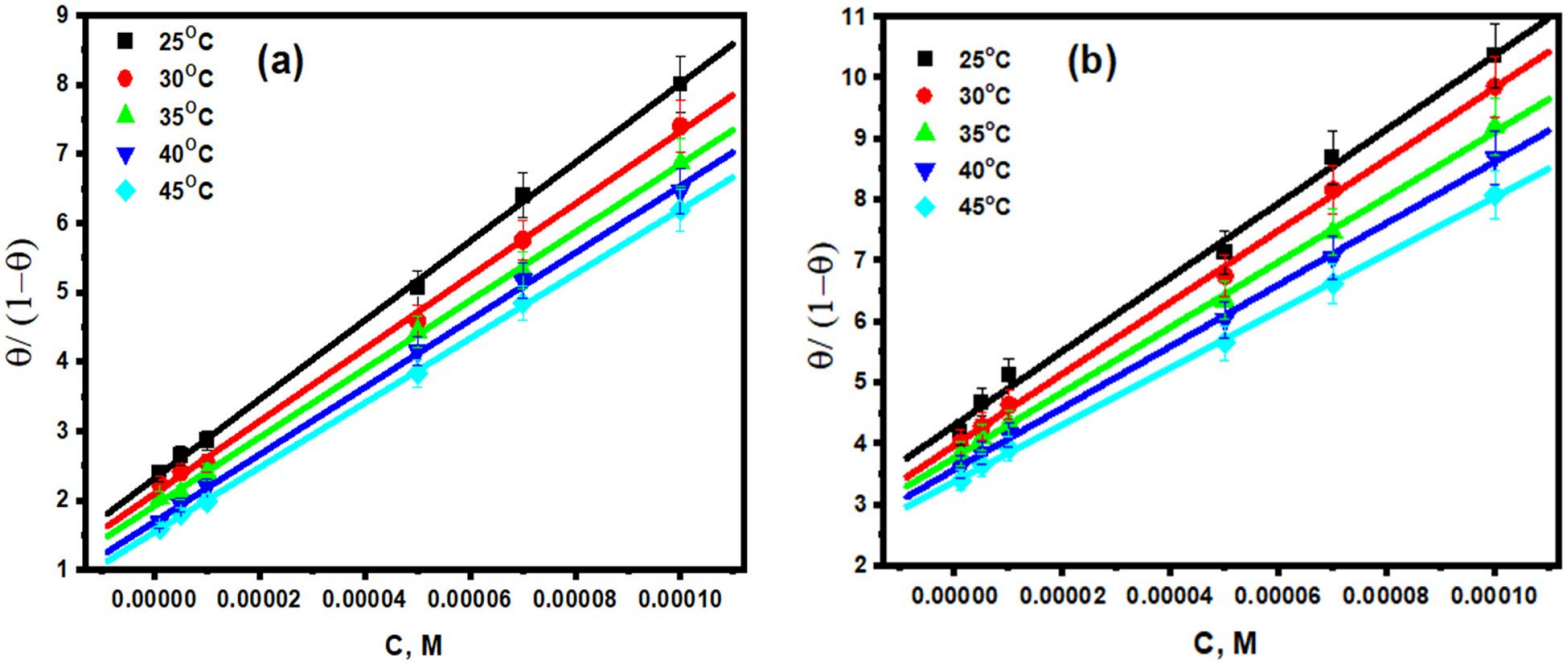
Langmuir adsorption isotherms of (**a**) DBPD and (**b**) DPAPD on the carbon steel surface in 1.0 M HCl at different temperatures.

Figure 2 illustrates the relationship between the term (θ/1 − θ) and the inhibitor concentration (C) for all investigated systems. The resulting plots display a high degree of linearity over the studied concentration range, with correlation coefficients (R^2^) exceeding 0.994 in all cases.

This excellent linear fit confirms that the adsorption of the investigated inhibitors on the carbon steel surface obeys the Langmuir adsorption isotherm. Such behavior suggests that the adsorption process involves the formation of a uniform monolayer of inhibitor molecules on the metal surface, with negligible interactions between adjacent adsorbed species.

The standard free energy of adsorption ΔG°_ads_ was calculated using the following relation^40^:

8 $${\mathrm{K}}_{{{\mathrm{ads}}}} = {1}/{55}.{\text{5 e}}^{{( - \Delta {\mathrm{G}}^\circ \;_{\mathrm{ads}}/{\mathrm{RT}})}}$$

where 55.5 is the molar concentration of water in the solution (in mole per liter), R is the universal gas constant, and T is the absolute temperature.

The adsorption equilibrium constant (K_ads_) provides a quantitative measure of the interaction strength between inhibitor molecules and the carbon steel surface. Higher K_ads_ values indicate stronger adsorption affinity and the formation of a more stable and protective film on the metal surface. In the present study, the relatively high Kads values obtained for both DBPD and DPAPD suggest a strong tendency of these molecules to adsorb spontaneously onto the steel surface, resulting in effective surface coverage even at low concentrations.

Furthermore, K_ads_ is directly related to the standard free energy of adsorption (ΔG°ads), reflecting the ability of inhibitor molecules to displace water molecules from the metal surface and stabilize the adsorbed layer. The higher K_ads_ observed for DPAPD compared to DBPD is consistent with its superior inhibition efficiency, as confirmed by electrochemical measurements and surface characterization techniques, indicating a direct correlation between adsorption strength and corrosion inhibition performance.

A deviation of the slope from unity suggests that the adsorption process does not strictly follow the ideal Langmuir model. This behavior may be attributed to lateral interactions between adsorbed inhibitor molecules on the carbon steel surface. In addition, variations in adsorption enthalpy with surface coverage may also contribute to this non-ideal behavior, as such effects are not accounted for in the classical Langmuir assumptions.

The calculated negative values of ΔG°_ads_ for DBPD and DPAPD confirm the spontaneous nature of adsorption and the formation of a thermodynamically stable protective layer on the metal surface. Although ΔG°_ads_ is often used as an indicator of adsorption strength, recent studies have shown that it should not be used as a sole criterion to distinguish between physisorption and chemisorption, as adsorption at metal–solution interfaces is inherently complex^41^.

Accordingly, the adsorption process in the present study is better described as a mixed-type mechanism involving both electrostatic interactions (physisorption) and charge transfer interactions (chemisorption), as supported by potentiodynamic polarization, EIS, surface characterization, and quantum chemical calculations.

In the present study, the ΔG°_ads_ values for both DBPD and DPAPD fall within the range of approximately − 37.0 to − 39.0 kJ mol⁻^1^. This intermediate range suggests that the adsorption mechanism involves a combined contribution of both physical and chemical interactions, reflecting a mixed physisorption–chemisorption mode.

Furthermore, the adsorption enthalpy was estimated using the Van’t Hoff equation^40^:

9 $$logK_{ads}= \left( { - \Delta {\mathrm{H}}^\circ_{{{\mathrm{ads}}}} /{\mathrm{2.303RT}}} \right) + {\mathrm{const}}$$

which describes how the adsorption equilibrium constant varies with temperature and allows the determination of the corresponding thermodynamic parameters^42^.

**Fig. 3:**
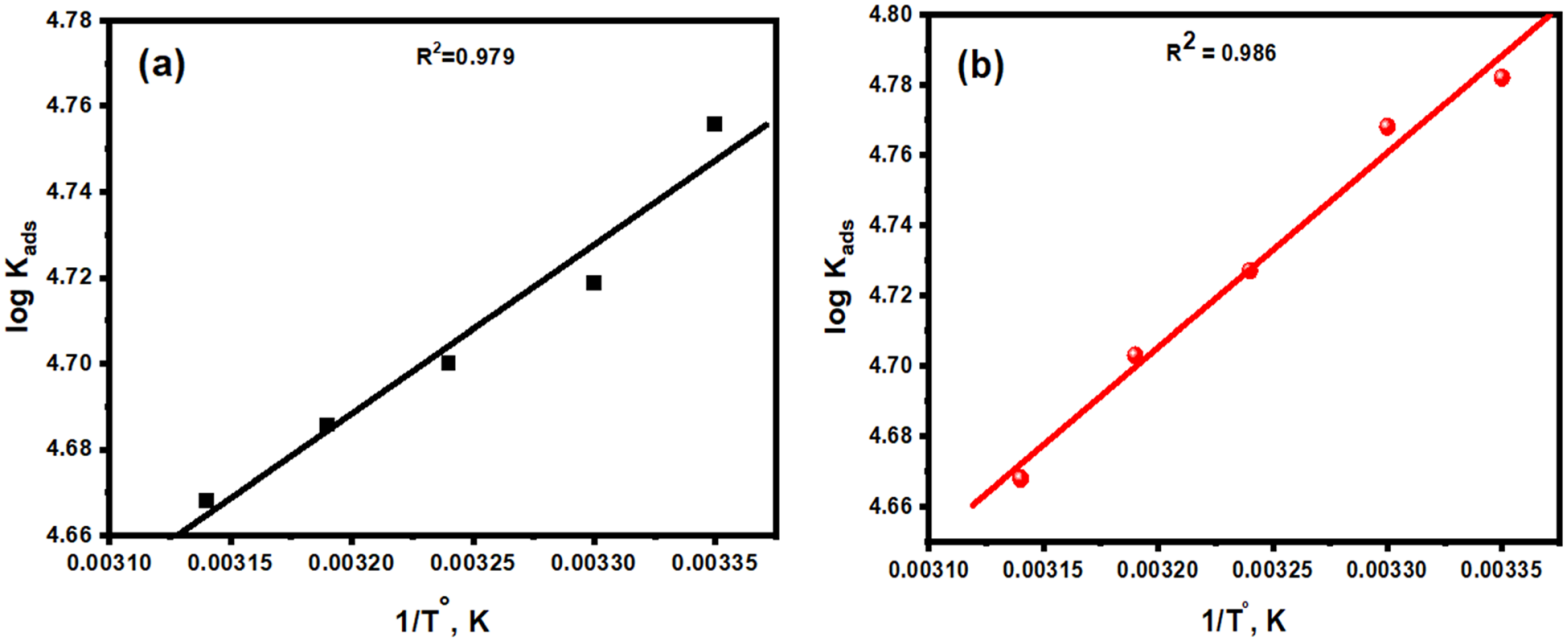
Log K_ads_ vs. (1/T) curves for carbon steel dissolution in 1.0 M HCl in the presence of (**a**) DBPD and (**b**) DPAPD.

Figure 3 presents the Van’t Hoff plots of log K_ads_ versus 1/T for DBPD and DPAPD, which were constructed to evaluate the temperature dependence of the adsorption process and to determine the thermodynamic parameters of adsorption. These plots provide important insight into the nature and strength of the interaction between the inhibitor molecules and the carbon steel surface. The linear behavior observed in all cases confirms that the adsorption process follows the Van’t Hoff relationship over the studied temperature range. The slopes of these straight lines were used to calculate the standard enthalpy of adsorption (ΔH°_ads_), while the intercepts were employed to derive additional thermodynamic parameters. The significance of this figure lies in its ability to correlate adsorption strength with temperature, thereby explaining the experimentally observed decrease in inhibition efficiency at elevated temperatures. The negative ΔH°_ads_ values obtained from these plots further confirm the exothermic nature of adsorption, indicating that the adsorption process becomes less favorable as temperature increases. This behavior supports the formation of a physically stable adsorbed layer with partial chemisorption contribution, consistent with the overall mixed adsorption mechanism proposed in this study.

As listed in Table 3, the calculated ΔH°_ads_ values are negative for both inhibitors, indicating that the adsorption process is exothermic. This suggests that the interaction between the inhibitor molecules and the metal surface becomes less favorable at higher temperatures. Consequently, an increase in temperature leads to a reduction in the extent of adsorption, which is in good agreement with the experimentally observed decrease in inhibition efficiency at elevated temperatures.

**Table 3:** Thermodynamic parameters for the adsorption of inhibitors on the carbon steel surface in 1.0 M HCl at different temperatures.

	DBPD				DPAPD			
Temp (K)	K_ads_ × 10^4^ M^−1^	-ΔG°_ads_ (kJ mol^−1^)	-ΔS°_ads_ (J mol^−1^k^−1^)	− ΔH°_ads_ (kJ mol^−1^)	K_ads_ × 10^4^M^−1^	− ΔG°_ads_ (kJ mol^−1^)	− ΔS°_ads_ (J mol^−1^k^−1^)	− ΔH°_ads_ (kJ mol^−1^)
298	5.699	37.064	113.38	3.265	6.065	37.218	109.47	4.595
303	5.233	37.471	112.86		5.861	37.756	109.44	
308	4.931	37.937	112.54		5.338	38.14	108.91	
313	4.85	38.510	112.57		5.047	38.613	108.68	
318	4.65	39.018	112.40		4.656	39.017	108.24	

In addition, the standard entropy of adsorption (ΔS°_ads_) was evaluated using the following thermodynamic equation^40^:

10 $$\Delta {\mathrm{S}}^\circ_{{{\mathrm{ads}}}} = (\Delta {\mathrm{H}}^\circ_{{{\mathrm{ads}}}} - \Delta {\mathrm{G}}^\circ_{{{\mathrm{ads}}}} /{\mathrm{T}})$$

The values of the standard entropy of adsorption (ΔS°_ads_) listed in Table 3 are negative for both inhibitors, indicating that the adsorption process is associated with a decrease in system entropy. This behavior suggests that the overall system becomes more ordered as the inhibitor molecules transition from the bulk solution to the metal surface and form an adsorbed layer. The reduction in entropy also implies that adsorption becomes less favorable at elevated temperatures, which is consistent with the observed decline in inhibition efficiency.

This phenomenon can be explained based on a quasi-substitution adsorption mechanism, in which inhibitor molecules in solution [Org(sol)] displace pre-adsorbed water molecules from the metal surface^43,44^. In this process, water molecules are released into the bulk phase, while inhibitor molecules occupy active sites on the carbon steel surface. The desorption of water molecules is typically associated with an increase in entropy and an endothermic contribution, whereas the adsorption of relatively larger organic molecules leads to a decrease in entropy due to restricted molecular motion and the formation of a more ordered interfacial structure, accompanied by an exothermic effect^45^.

Taken together, the evaluated thermodynamic parameters indicate that the adsorption of DBPD and DPAPD on the carbon steel surface is spontaneous, as confirmed by the negative ΔG°_ads_ values, and exothermic in nature, as reflected by the negative ΔH°_ads_ values. The negative ΔS°_ads_ values further demonstrate the formation of an ordered adsorbed layer at the metal/solution interface. These findings support a mixed adsorption mechanism, in which both physisorption and chemisorption contribute cooperatively to the overall inhibition performance and the stability of the protective film.

### Kinetic—thermodynamic corrosion parameters

The corrosion inhibition mechanism and adsorption behavior of the synthesized inhibitors were further investigated through thermodynamic and kinetic–thermodynamic analyses. The corrosion performance of carbon steel in 1.0 M HCl solution was evaluated in both the absence and presence of various concentrations of the studied inhibitors.

To gain deeper insight into the influence of the inhibitors on the corrosion process, the apparent activation energy (Eₐ*) was determined. This parameter provides valuable information regarding the energy barrier associated with the corrosion reaction. The activation energy values were calculated using the Arrhenius equation (Eq. 11)^46^.

11 $$k_{corr} = {\mathrm{Ae}}^{{ - {\mathrm{Ea}}*/{\mathrm{RT}}}}$$

where k_corr_ is the corrosion rate, *A* is the Arrhenius pre-exponential factor, * E*_*a*_ is the apparent activation energy, *R* is the universal gas constant, and *T* is the absolute temperature.

**Fig. 4:**
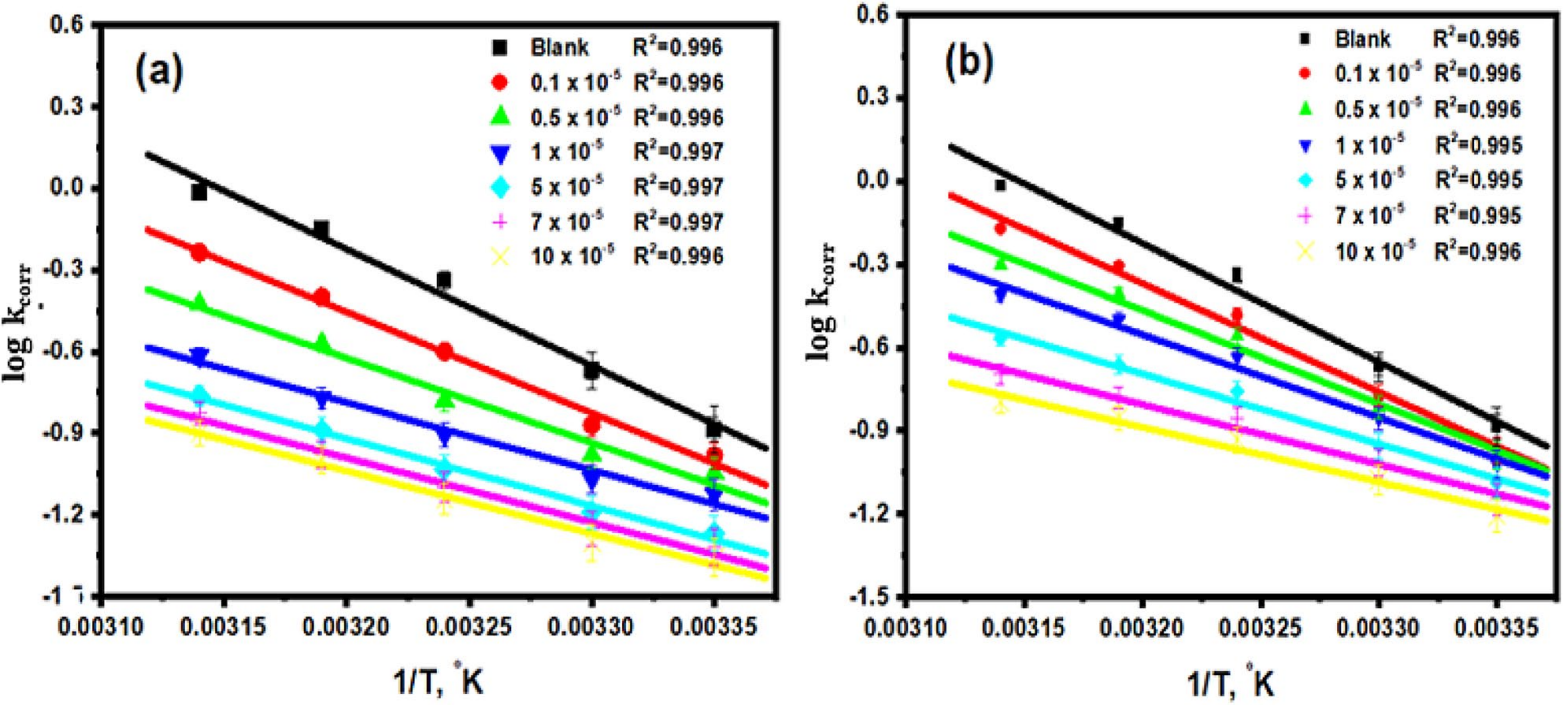
Arrhenius plots for carbon steel corrosion rates (*k*_corr_.) after 180 min of immersion in 1.0 M HCl in the absence and presence of different concentrations of (**a**) DBPD and (**b**) DPAPD.

A linear relationship was obtained from the plot of log *k*_corr_ versus 1/T (Fig. 4), indicating that the corrosion process of carbon steel in 1.0 M HCl follows Arrhenius-type behavior. The apparent activation energy (Eₐ*) values were calculated from the slopes of these linear plots and are summarized in Table 4. The calculated activation energy (Eₐ*) values clearly indicate an increase in the presence of the investigated inhibitors compared to the uninhibited system. Specifically, the Eₐ* value for the blank solution (1.0 M HCl) is 25.87 kJ mol⁻^1^, whereas it increases to 34.51 kJ mol⁻^1^ for DBPD and 35.58 kJ mol⁻^1^ for DPAPD at the highest studied concentration. This increase confirms that the presence of inhibitor molecules introduces an additional energy barrier that retards the corrosion process by blocking active sites on the carbon steel surface and reducing the rate of metal dissolution.

**Table 4:** Activation parameters for the dissolution of carbon steel in the presence and absence of different concentrations of inhibitors in 1.0 M HCl.

	DBPD			DPAPD		
Conc (M)	Ea* (kJ mol^−1^)	ΔH* (kJ mol^−1^)	− ΔS* (J mol^−1^k^−1^)	Ea* (kJ mol^−1^)	ΔH* (kJ mol^−1^)	− ΔS* (J mol^−1^k^−1^)
Blank (1.0 M HCl )	25.87	92.10	239.12	25.87	92.1	239.12
0.1 × 10^-5^	27.12	82.40	244.31	27.91	87.5	242.50
0.5 × 10^-5^	29.55	70.10	248.31	30.86	77.3	245.89
1 × 10^-5^	30.81	61.04	252.12	31.12	67.4	248.70
5 × 10^-5^	31.63	60.90	254.01	31.97	59.4	251.73
7 × 10^-5^	32.83	54.90	256.44	32.37	53.6	253.84
10 × 10^-5^	35.58	52.80	258.09	34.51	51.2	256.10

The increase in activation energy upon addition of the inhibitors suggests the introduction of an additional energy barrier that retards the corrosion reaction, thereby reducing the rate of metal dissolution. In general, higher Eₐ* values are associated with a decrease in corrosion rate and reflect improved inhibition efficiency^47–51^.

Although activation energy alone does not provide a complete description of the adsorption mechanism, its increase in the presence of inhibitors supports the hypothesis that these molecules adsorb onto the metal surface, blocking active sites and hindering the charge transfer processes involved in corrosion.

**Fig. 5:**
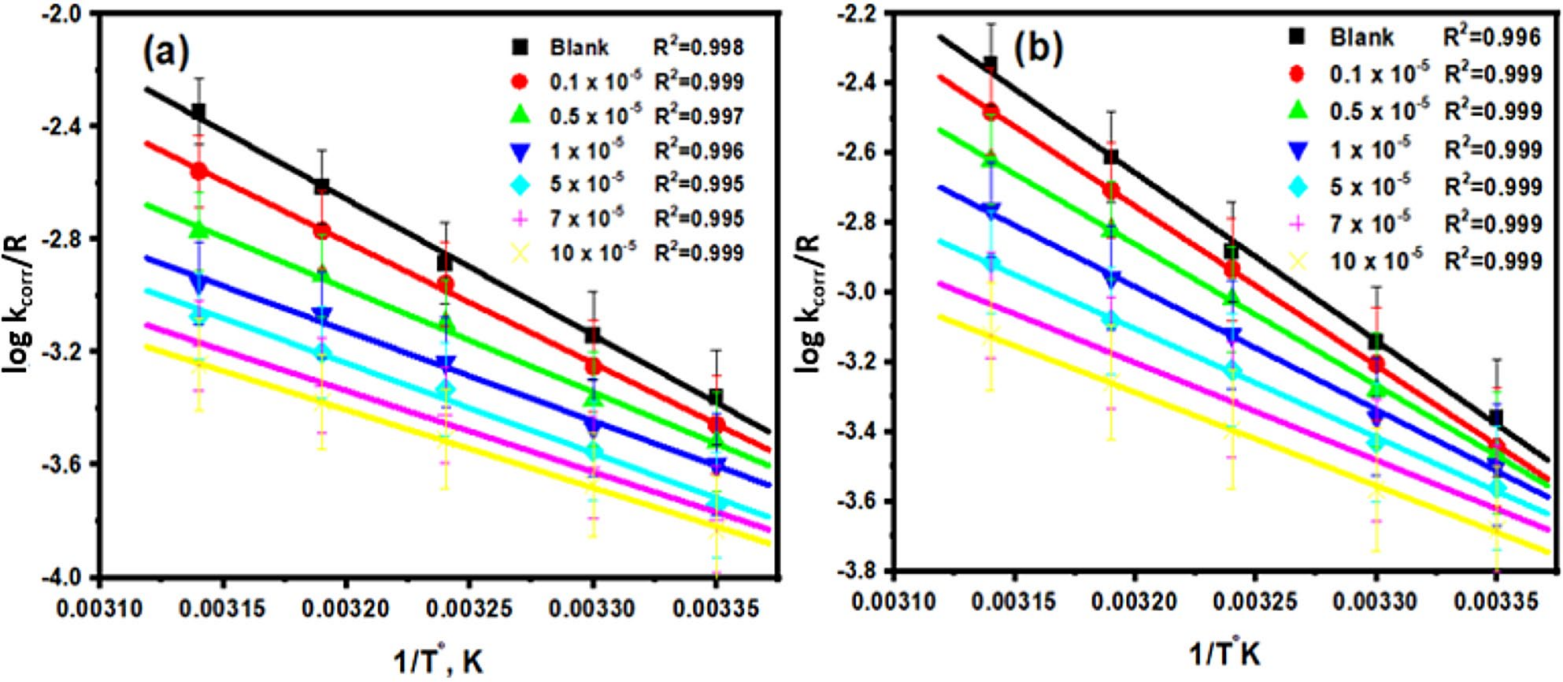
Plots of (log*k*_corr_) vs.1/T for corrosion of C-steel in 1.0 M HCl in the absence and presence of different concentrations of (**a**) DBPD and (**b**) DPAPD.

To further elucidate the corrosion mechanism, the activation enthalpy (ΔH*) and activation entropy (ΔS*) were evaluated based on transition state theory using the following equation (Eq. 12)^52,53^:

12 $${\mathrm{K}} = {\mathrm{RT}}/{\mathrm{Nh}} {\mathrm{e}}^{{\left( {\Delta {\text{S }}*/{\mathrm{R}}} \right)}} {\mathrm{e}}^{{( - }{\Delta {\text{H }}*/{\text{ RT }})}}$$

where *h* is Planck’s constant and *N* is Avogadro’s number. A linear relationship was obtained by plotting log(k/T) versus 1/T (Fig. 5), yielding a straight line with a slope corresponding to (− ΔH*/2.303R) and an intercept equal to [log(RT/Nh) + (ΔS*/2.303R)]^54,55^.The activation parameters, including the activation enthalpy (ΔH*) and activation entropy (ΔS*), are summarized in Table 4. The data clearly show that the presence of DBPD and DPAPD leads to an increase in ΔH*, indicating that the corrosion process becomes energetically less favorable in the presence of the inhibitors. This behavior can be attributed to the formation of a strongly adsorbed and stable protective layer on the carbon steel surface, which effectively increases the energy barrier for metal dissolution.

Moreover, the negative values of ΔS* obtained for both uninhibited and inhibited systems suggest a reduction in the degree of randomness during the formation of the activated complex. This implies that the transition state is more ordered than the initial state, reflecting a more structured and constrained arrangement at the metal/solution interface.

Such characteristics are indicative of an associative-type mechanism, where the activated complex is formed through increased organization rather than bond cleavage. This interpretation is consistent with the adsorption of inhibitor molecules onto the carbon steel surface, resulting in the formation of a compact and adherent protective film.

The combined kinetic and thermodynamic analyses confirm that DBPD and DPAPD act as efficient corrosion inhibitors through a synergistic adsorption mechanism involving both physisorption and chemisorption. The formation of this protective adsorbed layer effectively blocks active corrosion sites and suppresses charge transfer reactions at the metal/solution interface^56–58^.

#### Proposed corrosion inhibition mechanism

The corrosion inhibition mechanism of DBPD and DPAPD in 1.0 M HCl solution can be rationalized in terms of surface charge distribution, electronic structure of the inhibitors, and metal–adsorbate interactions at the carbon steel/electrolyte interface. In aqueous acidic medium, the steel surface becomes negatively polarized due to specific adsorption of chloride ions, creating an electrochemically favorable interface for the adsorption of cationic inhibitor species.

Upon immersion, DBPD and DPAPD molecules undergo preferential protonation at the N,N-dimethylamino group, which represents the most basic and electron-rich site in the molecular structure, leading to the formation of ammonium species. This protonation enhances the ionic character of the molecules and facilitates their initial adsorption onto the metal surface through strong electrostatic interactions (physisorption) with pre-adsorbed chloride ions, resulting in rapid surface coverage.

**Fig. 6:**
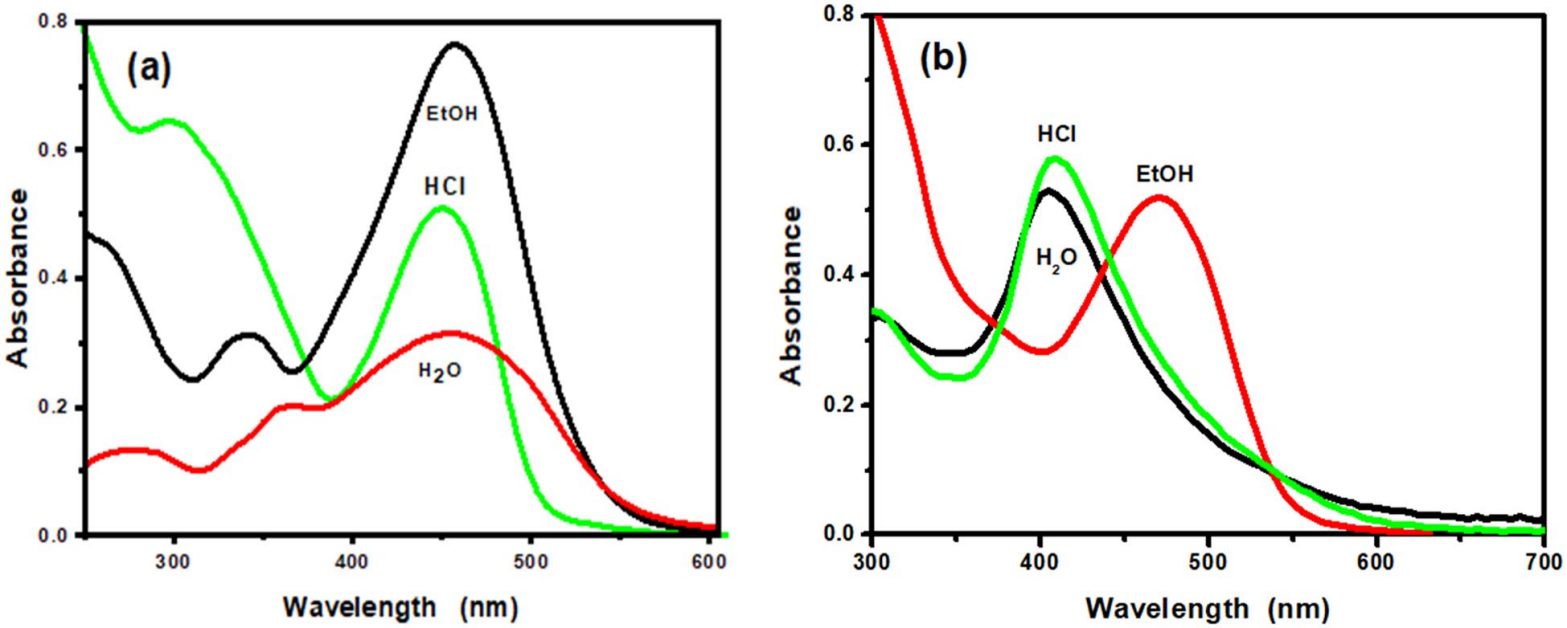
Electronic absorption spectra of DBPD and DPAPD in EtOH-prepared solution, aqueous medium H₂O and 1.0 M HCl solution.

The electronic nature of the inhibitors was further supported by UV–Vis spectral analysis in different media (aqueous and acidic conditions), where a slight hypsochromic shift was observed for both DBPD and DPAPD as shown in Fig. 6. This weak spectral variation indicates only minor perturbation of the electronic structure, suggesting that the conjugated π-system remains largely preserved upon transition to the corrosion medium. Such behavior is consistent with protonation predominantly occurring at the N,N-dimethylamino group, without significant disruption of the chromophoric framework, while protonation at the carbonyl oxygen is less favorable under these conditions.

As adsorption progresses, a more stable interaction is established via chemisorption, involving coordination between lone pairs of heteroatoms (particularly carbonyl oxygen) and the delocalized π-electron system with vacant d-orbitals of surface iron atoms, leading to Fe–O coordination bonds and Fe–π interactions. Possible back-donation from filled d-orbitals of iron into antibonding orbitals of the inhibitor further stabilizes the adsorbed layer.

**Scheme 1:**
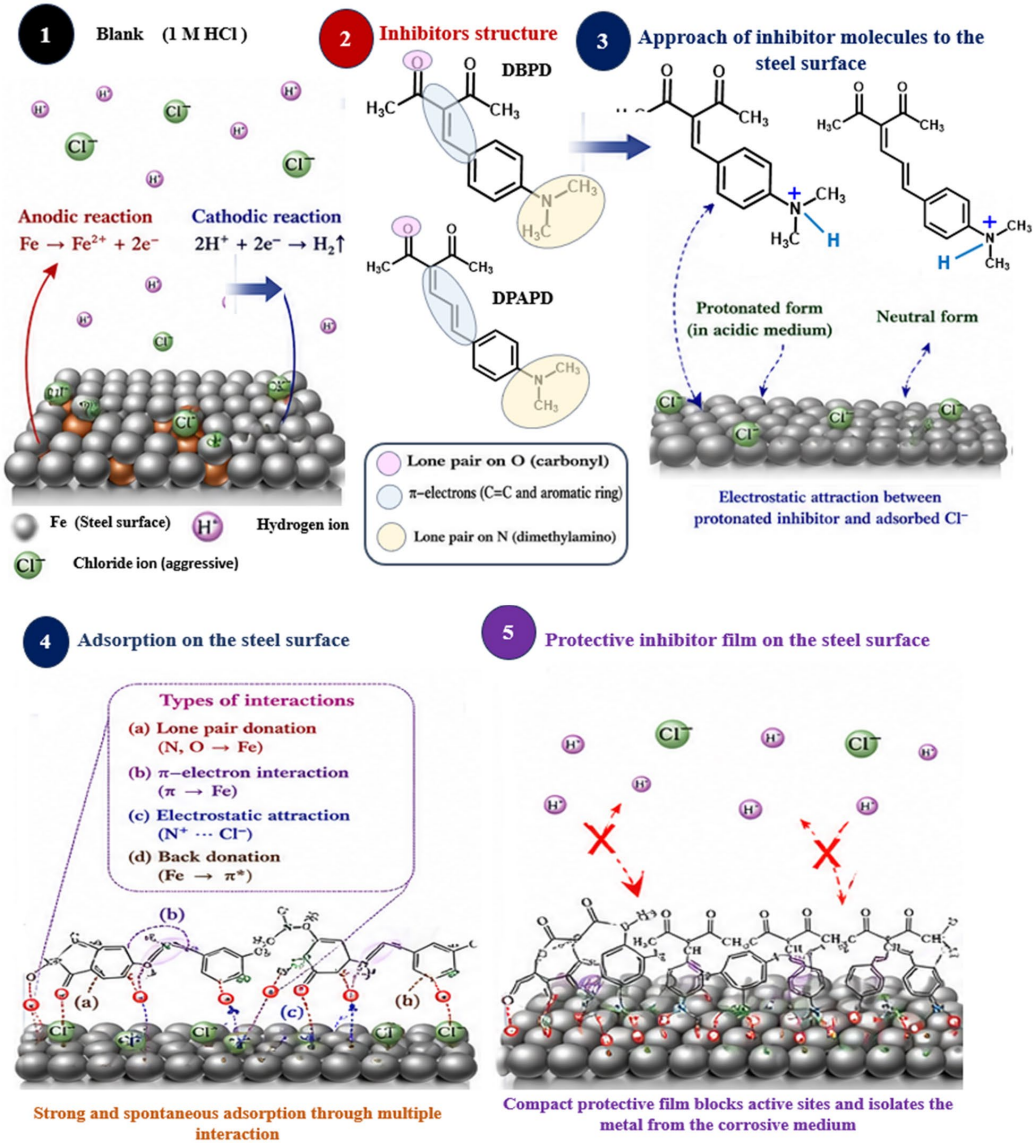
Proposed adsorption and corrosion inhibition mechanism of DPAPD/DBPD.

From a molecular perspective, the higher inhibition efficiency of DPAPD compared to DBPD can be attributed to its more extended π-conjugation and increased electron density, which enhance polarizability and strengthen metal–inhibitor interactions. Consequently, DPAPD forms a more compact and stable adsorbed film with higher surface coverage (θ), effectively blocking active corrosion sites and suppressing both anodic metal dissolution and cathodic hydrogen evolution reactions (Scheme 1).

### Potentiodynamic polarization measurements

**Fig. 7:**
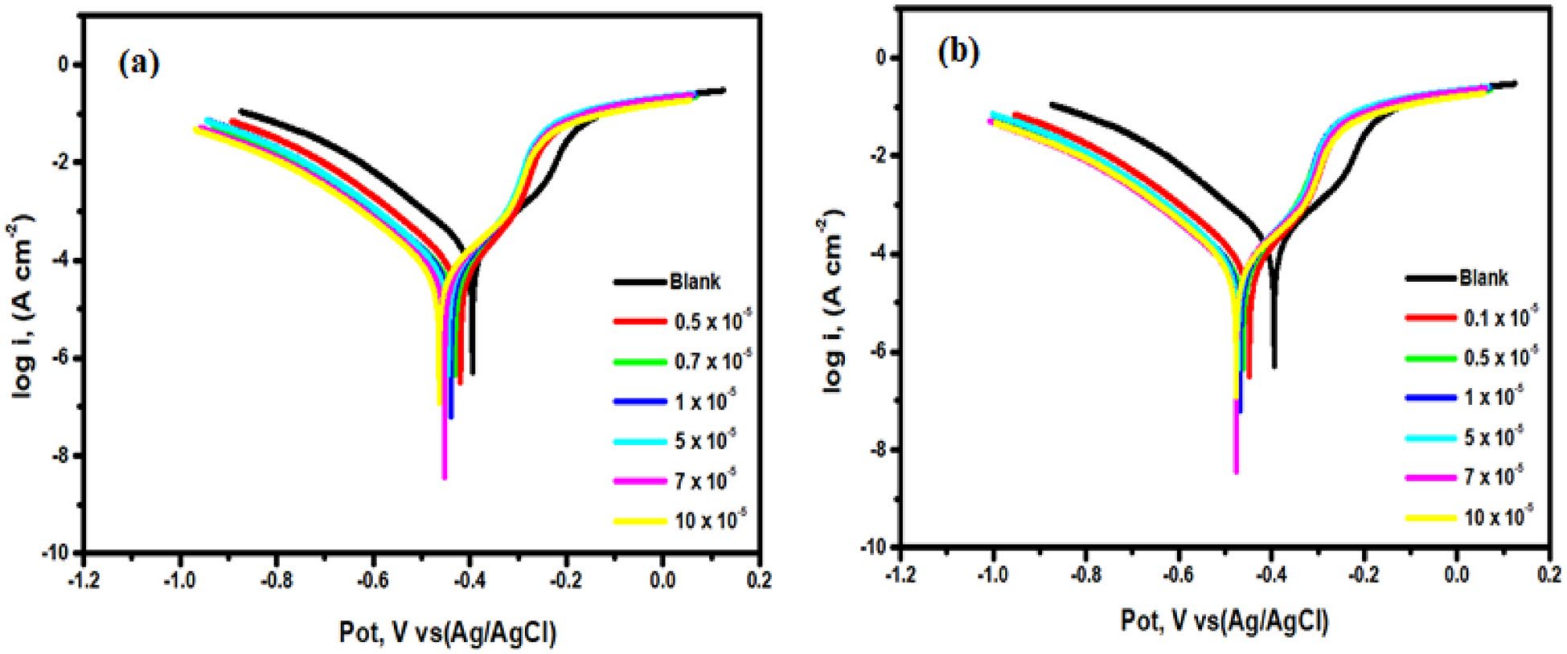
Potentiodynamic polarization curve plots for corrosion of carbon steel in 1.0 M HCl of the inhibitors (**a**) DBPD and (**b**) DPAPD at different concentrations (0.1 × 10^–5^ to 10 × 10^–5^ M) at 25 °C.

The potentiodynamic polarization curves for carbon steel in 1.0 M HCl solution, recorded in the absence and presence of DBPD and DPAPD at 25 °C, are presented in Fig. 7a–b. The corresponding electrochemical parameters, including the corrosion current density (i_corr_), corrosion potential (E_corr_, mV vs Ag/AgCl), and the anodic and cathodic Tafel slopes (β_a_ and β_c_), were extracted from the polarization profiles.

The introduction of the inhibitors results in a significant decrease in both anodic and cathodic current densities, indicating a substantial reduction in the overall corrosion rate. This behavior demonstrates that DBPD and DPAPD effectively suppress both the anodic metal dissolution and the cathodic hydrogen evolution reactions, confirming their mixed-type inhibitory nature in acidic media.

As summarized in Table 5, the corrosion current density (i_corr_) decreases markedly upon addition of the inhibitors, from 217.59 μA cm⁻^2^ for the uninhibited solution to 27.61 μA cm⁻^2^ in the presence of DBPD and further to 21.18 μA cm⁻^2^ for DPAPD. This pronounced reduction reflects the formation of an adsorbed protective film that limits the accessibility of aggressive species, such as chloride ions, to the metal surface.

**Table 5:** Effect of concentration of the investigated compounds on the free corrosion potential (E_corr_, mV vs Ag/AgCl), corrosion current density (i_corr_), Tafel slopes (βa and βc), corrosion rate (C.R.), degree of surface coverage θ, and inhibition efficiency (%IE) for the corrosion of carbon steel in 1.0 M HCl at 25 °C.

Inhibitor	Conc (M)	− E_corr_ (mV, vs Ag/AgCl)	i_corr_ (μA cm^2^)	β_c_ (mV dec^−1^)	β_a_ (mV dec^−1^)	corrosion rate (CR) (mp y^−1^)	Ɵ	%IE
DBPD	Blank (1.0 M HCl )	394	217.59	142.31	128.12	252.03	–	–
	0.1 × 10^–5^	438	73.91	126.19	117.02	92.64	0.660	66.03
	0.5 × 10^–5^	444	61.96	118.02	109.58	72.68	0.715	71.52
	1 × 10^–5^	449	49.93	104.96	104.96	58.58	0.770	77.04
	5 × 10^–5^	453	38.12	98.71	115.98	38.13	0.824	82.48
	7 × 10^–5^	458	31.54	79.61	111.43	27.18	0.855	85.50
	10 × 10^–5^	463	27.61	69.14	110.33	19.56	0.873	87.31
DPAPD	Blank (1.0 M HCl )	394	217.59	142.31	128.12	252.03	–	–
	0.1 × 10^–5^	445	51.12	123.09	139.67	102.45	0.765	76.50
	0.5 × 10^–5^	452	42.63	127.62	143.07	88.97	0.804	80.40
	1 × 10^–5^	467	38.17	113.77	126.64	69.14	0.824	82.45
	5 × 10^–5^	472	28.61	113.78	131.04	45.56	0.868	86.85
	7 × 10^–5^	474	25.84	110.22	161.23	36.12	0.881	88.12
	10 × 10^–5^	478	21.18	98.70	165.17	22.93	0.902	90.20

In terms of corrosion potential, a shift toward more negative E_corr_ values is observed upon inhibitor addition, with values ranging from − 394 to − 463 mV for DBPD and from − 394 to − 474 mV vs Ag/AgCl for DPAPD. The corrosion potential (E_corr_) exhibits only a moderate shift upon addition of DBPD and DPAPD. However, it is important to note that the classification of inhibitors as anodic, cathodic, or mixed-type cannot be reliably based solely on the magnitude of E_corr_ displacement. Instead, it should be supported by a comprehensive evaluation of polarization behavior, including changes in both anodic and cathodic Tafel slopes, as well as the reduction in corrosion current density.

In the present study, the relatively small shifts in E_corr_, together with the simultaneous decrease in both anodic and cathodic current densities, indicate that the investigated compounds inhibit both metal dissolution and hydrogen evolution reactions. This confirms their mixed-type inhibition behavior in 1.0 M HCl solution^59,60^.

Moreover, the relatively small variations in the Tafel slopes (β_a_ and β_c_) suggest that the fundamental electrochemical mechanisms of the anodic and cathodic reactions remain largely unchanged. This indicates that the inhibitors primarily act by reducing the active surface area through adsorption and blocking of reactive sites, rather than altering the reaction pathways.

The inhibition efficiencies calculated from polarization measurements reach maximum values of 87.31% for DBPD and 90.2% for DPAPD at the highest studied concentration. These values are in good agreement with those obtained from electrochemical impedance spectroscopy (EIS), confirming the reliability of the results. The superior performance of DPAPD can be attributed to its stronger adsorption affinity and its ability to form a more compact and stable protective layer on the carbon steel surface.

### Electrochemical impedance spectroscopy

**Fig. 8:**
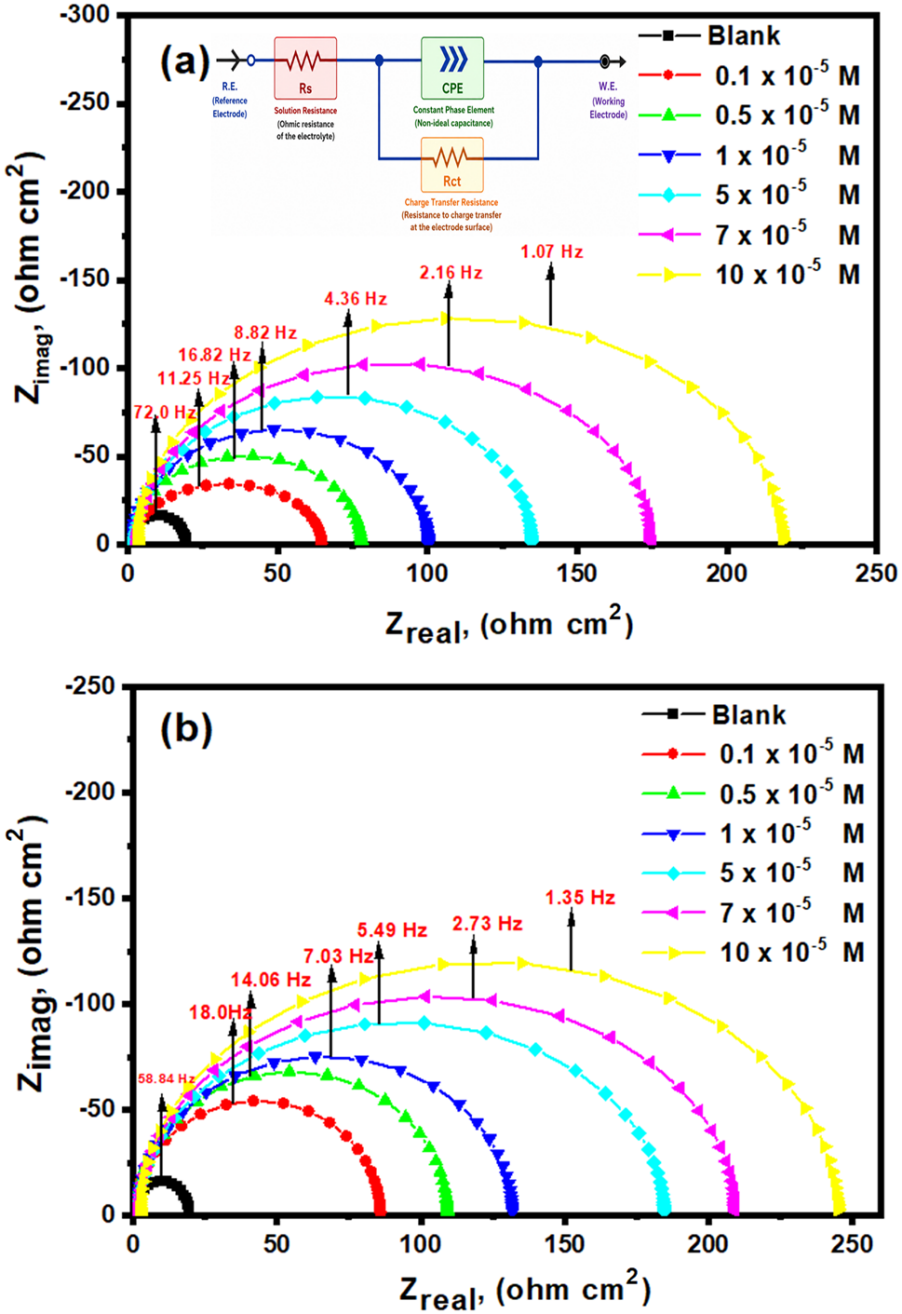
Nyquist diagrams of carbon steel in 1.0 M HCl of the inhibitors (**a**) DBPD and (**b**) DPAPD at different concentrations (0.1** × **10^–5^ to 10 × 10^–5^ M) at 25 °C. Inset : The electrical equivalent circuit model used to fit the results (**c**).

**Fig. 9:**
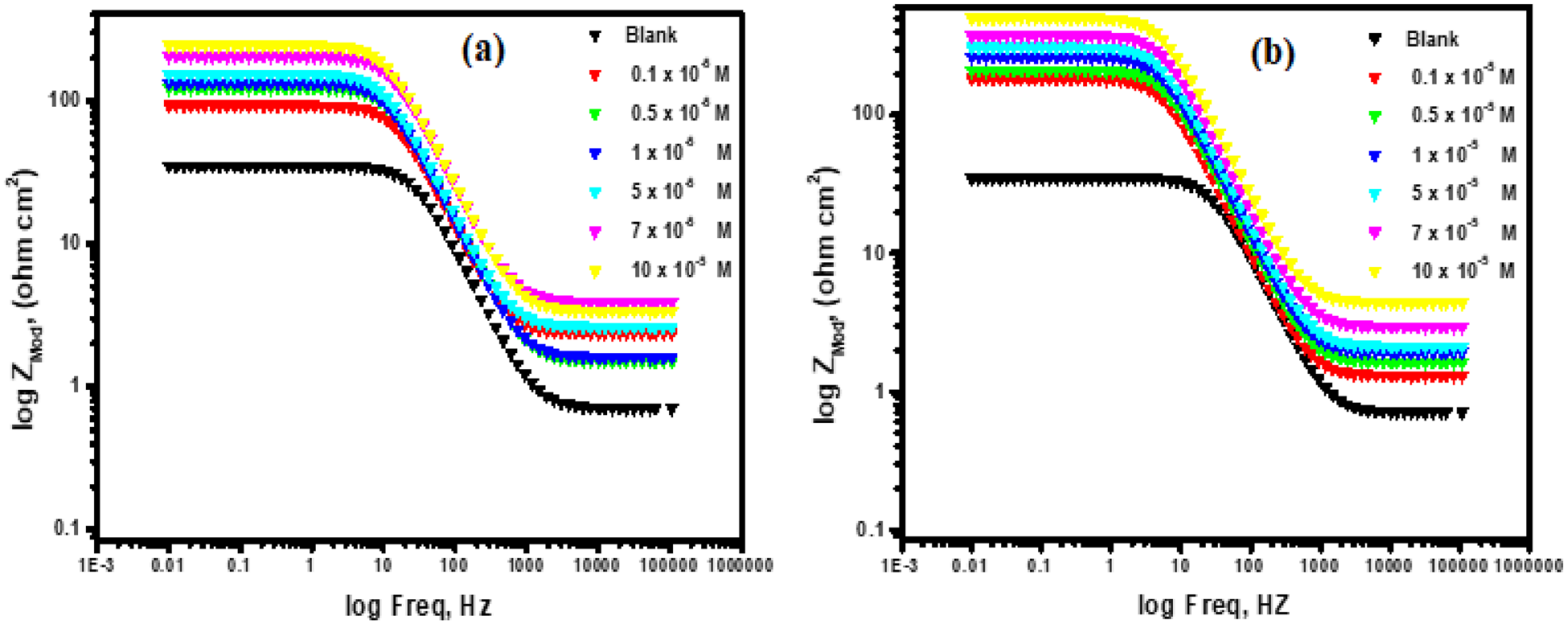
The Bode plots for corrosion of carbon steel in 1.0 M HCl of the inhibitors (**a**) DBPD and (**b**) DPAPD at different concentrations (0.1 × 10^–5^ to 10 × 10^−5 ^M) at 25 °C.

**Fig. 10:**
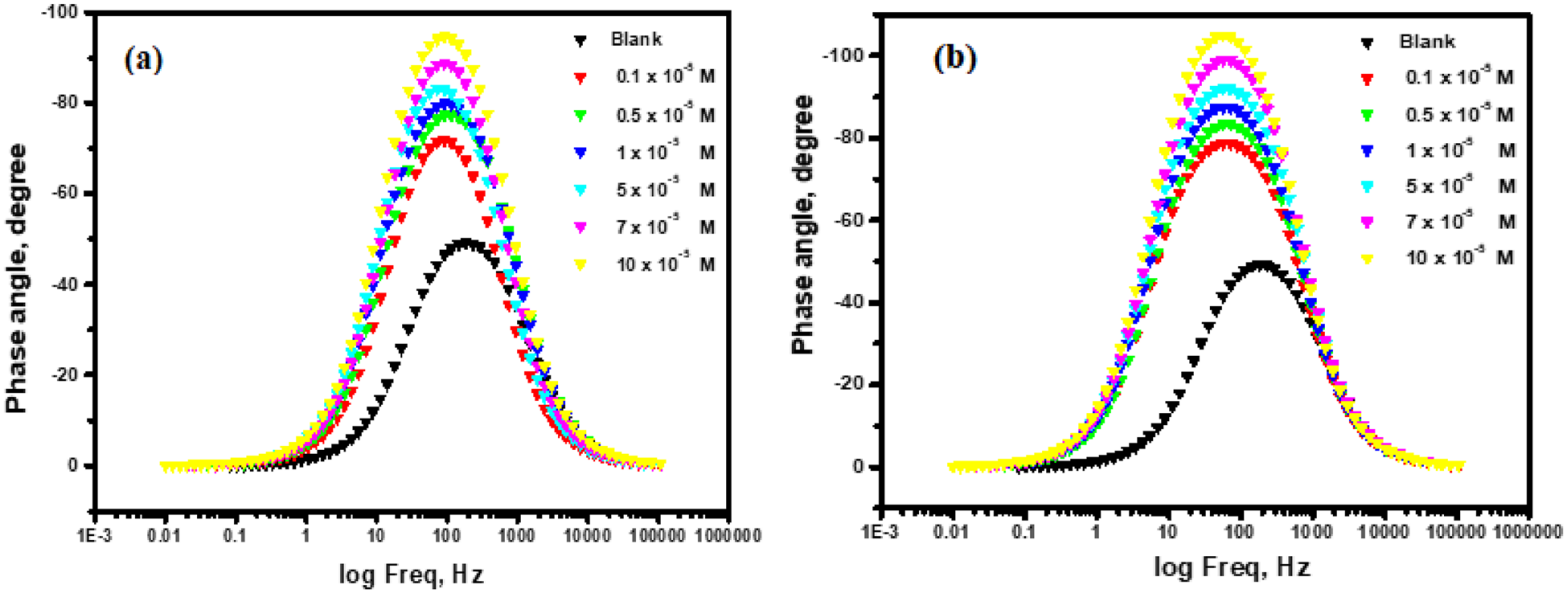
Phase angle for corrosion of carbon steel in 1.0 M HCl of the inhibitors (**a**) DBPD and (**b**) DPAPD at different concentrations (0.1 × 10^–5^ to 10 × 10^–5^ M) at 25 °C.

Electrochemical impedance spectroscopy (EIS) measurements were conducted to investigate the corrosion behavior of carbon steel in 1.0 M HCl solution at 298 K, in both the absence and presence of different concentrations of DBPD and DPAPD. The impedance responses are presented in Figs. 8, 9 and 10, including Nyquist plots (Fig. 8a–b), Bode magnitude plots (Fig. 9), and Bode phase angle plots (Fig. 10).

The Nyquist plots exhibit single depressed capacitive semicircles, indicating that the corrosion process is predominantly governed by charge transfer at the metal/solution interface. The depressed nature of the semicircles reflects surface heterogeneity, which may arise from factors such as surface roughness and the non-uniform adsorption of inhibitor molecules.

The presence of a single time constant in the Bode phase angle plots further confirms that the fundamental corrosion mechanism remains unchanged upon addition of the inhibitors, with charge transfer continuing to be the controlling step^61^.

A progressive increase in the diameter of the Nyquist semicircles is observed with increasing concentrations of DBPD and DPAPD. This behavior corresponds to an increase in the charge-transfer resistance (R_ct_), indicating enhanced adsorption of inhibitor molecules on the carbon steel surface. Consequently, greater surface coverage is achieved, which reduces the accessibility of aggressive species and improves the corrosion resistance of the system^62,63^.

The Bode magnitude plots support these findings, showing a noticeable increase in impedance values, particularly at lower frequencies, with increasing inhibitor concentration. This trend is indicative of the formation of a more compact and protective adsorbed layer that effectively impedes electrochemical reactions at the interface.

In addition, the Bode phase angle plots reveal an increase in the maximum phase angle at intermediate frequencies upon addition of the inhibitors. This enhancement reflects improved capacitive behavior of the metal/solution interface, which is attributed to the formation of a stable and adherent protective film. Higher phase angle values are associated with a more ideal capacitive response and enhanced surface protection, thereby suppressing both anodic and cathodic reactions^64^.

Using the equivalent circuit depicted inset of Fig. 8, where Rs (the solution resistance), R_c__t_, Cdl, and the relaxation time (τ_R_) are documented in Table 6**,** the well-fitted data were gathered. A larger depression is seen in the Nyquist semicircle diagram due to the CPE, which is thought of as an irregular surface irregularity of the electrode and functions as a capacitor at the metal-solution interface”. While the Cdl values exhibit the opposite association^65^, the values of R_ct_ likewise increase as the inhibitor concentration rises. This idea may result from inhibitor adsorption on the metal surface or from H_2_O molecules desorbing from the CS surface^66^. It is evident from increasing the values of n (0.813–0.732), Table 6 for both DBPD and DPAPD, in comparison to the blank sample that the inhibitor uses adsorption to promote surface uniformity^67^. The continuous increase in reduced chi-squared values with concentration reflects a systematic deterioration in the goodness of fit of the ideal semicircle model. This behavior suggests a transition from near-ideal capacitive behavior at low concentrations to a more complex interfacial response at higher concentrations, characterized by increased deviation from ideal charge-transfer behavior and possible distribution of time constants.

**Table 6:** Electrochemical kinetic parameters obtained by the EIS technique in 1.0 M HCl without and with various concentrations of investigated compounds at 25 °C.

	DBPD								DPAPD							
Conc (M)	R_CT_ (Ω cm^2^)	n	Cdl µF/cm^2^	τ_Rms_	Ɵ	%IE	Mean ± SD	Chi-squared (χ^2^) × 10^–3^	R_CT_ (Ω cm^2^)	n	Cdl µF/cm^2^	τ_Rms_	Ɵ	%IE	Mean ± SD	Chi-squared (χ^2^)) × 10^–3^
Blank	19.70	0.732	376.74	7.42	–	–	− 3.9 2 ± 5.13	1.90	19.70	0.732	376.74	7.42	–	–	− 3.67 ± 5.04	1.92
0.1 × 10^–5^	64.40	0.756	252.09	16.24	0.694	69.4	− 7.71 ± 10.62	8.26	86.07	0.740	240.44	20.69	0.771	77.1	− 11.87 ± 16.62	19.54
0.5 × 10^–5^	78.10	0.757	249.67	19.50	0.747	74.77	− 11.25 ± 15.51	17.42	110.47	0.744	235.84	26.05	0.821	82.1	− 14.90 ± 20.85	30.82
1 × 10^–5^	99.70	0.758	228.17	22.75	0.802	80.24	− 14.61 ± 20.04	30.98	132.57	0.758	232.69	30.85	0.851	85.1	− 16.45 ± 23.03	37.48
5 × 10^–5^	134.70	0.76	224.59	30.25	0.853	85.37	− 18.69 ± 25.80	47.78	183.74	0.76	226.02	41.53	0.892	89.2	− 20.01 ± 27.97	55.88
7 × 10^–5^	174.69	0.771	211.42	36.93	0.887	88.72	− 22.92 ± 31.61	72.54	208.76	0.7629	223.34	46.62	0.905	90.5	− 22.69 ± 31.69	72.25
10 × 10^–5^	219.05	0.813	193.87	42.47	0.91	91.00	− 28.59 ± 39.45	112.01	244.78	0.766	222.75	54.52	0.919	91.9	− 26.25 ± 36.69	96.21

Table 7 provides a comprehensive comparative assessment of the corrosion inhibition performance of the synthesized bis-chalcone derivatives (DBPD and DPAPD) relative to a series of previously reported organic inhibitors for carbon steel in acidic media^68–73^. It is clearly evident that the present compounds exhibit markedly superior inhibition efficiencies at 10 × 10^–5^ M, surpassing most of the reported systems under comparable experimental conditions. As DPAPD demonstrates outstanding performance, achieving inhibition efficiencies exceeding 91% consistently across weight loss (WL), potentiodynamic polarization (PP), and electrochemical impedance spectroscopy (EIS) techniques. This level of agreement across multiple independent methods highlights the robustness and reliability of the obtained results. The superior inhibition behavior of DBPD and particularly DPAPD can be rationalized based on their molecular architecture. The presence of electron-donating dimethylamino groups, extended π-conjugation, and multiple active adsorption centers enhances electron density distribution and facilitates strong donor–acceptor interactions with the Fe surface. Additionally, the planar structure of the bis-chalcone framework promotes effective surface coverage and stable film formation, leading to improved barrier properties against aggressive ions.

**Table 7:** Comparison of the as-synthesized DBPD and DPAPD inhibition effectiveness with that of a few other approved inhibitors for the carbon steel electrode under various corrosive conditions.

No	Inhibitor	Corrosive media	Technique	Optimum concentration	%IE	Ref
1	N(Butylidene)-5-(3,3-Dimethyltriaz-1-en-1-yl)-1H-Imidazole-4-Carboxamide (BDIC)	1 × 10^–4^ M	EIS PP	1 M HCl	76.5 75.7	^68^
2	1,3-diphenylprop-2-en-1-one oxime (CO) 4-nitrophenyl-1-phenylprop-2-en-1-one(CO-NO_2_)	4.48 × 10^–4^ M	WL PP EIS	1 M HCl	67.0 77.0 78.0	^69^
			WL PP EIS		65.0 72.0 70.0	
3	N’-furan-2-yl-methylene-hydrazine carbodithioic acid N’-(4-dimethylamino-benzylidene)-hydrazine carbodithioic acid N’-(3-nitro-benzylidene)-hydrazine carbodithioic	1 × 10^-4^ M	EIS PP WL	0.5 M HCl	40.2 47.36 47.0	^60^
			EIS PP WL		65.20 79.04 48.0	
			EIS PP WL		80.2 91.65 86.0	
4	Chalcone oxime derivatives -CO–H, -CO-OMe	1.12 × 10^–4^ M	WL PP EIS	0.5 M H_2_SO_4_	58.0 63.0 68.0	^71^
			WL PP EIS		70.0 71.0 74.0	
5	(E)-ethyl 2-(4-(3-(4-fluorophenyl)acryloyl)phenoxy)acetate	1.0 × 10^–4^ M	WL PP EIS	1 M HCl	76.0 76.0 66.0	^72^
6	(E)-5-(4-(dimethylamino)phenyl)-3-(4-(dimethylamino)styryl)-4,5-dihydro-1H-pyrazole-1-carboxamide (DPC-2) 5-(4-(dimethylamino)phenyl)-3-phenyl-4,5-dihydro-1H-pyrazole-1-carboxamide (DPC-1)	1.6 × 10^–4^ M	WL PP EIS	1 M HCl	66.74 72.11 61.68	^73^
		2.0 × 10^–4^ M	WL PP EIS		57.82 30.75 27.30	
7	3-(4-(dimethylamino)benzylidene)pentane-2,4-dione (**DBPD)** 3-(3-(4-dimethylamino)phenylallylidene)pentane-2,4-dione** (DPAPD)**	10 × 10^–5^ M	WL PP EIS	1 M HCl	88.9 87.31 91.0	This work
			WL PP EIS		91.2 90.2 91.1	

### Surfae examination

#### Scanning electron microscopy

**Fig. 11:**
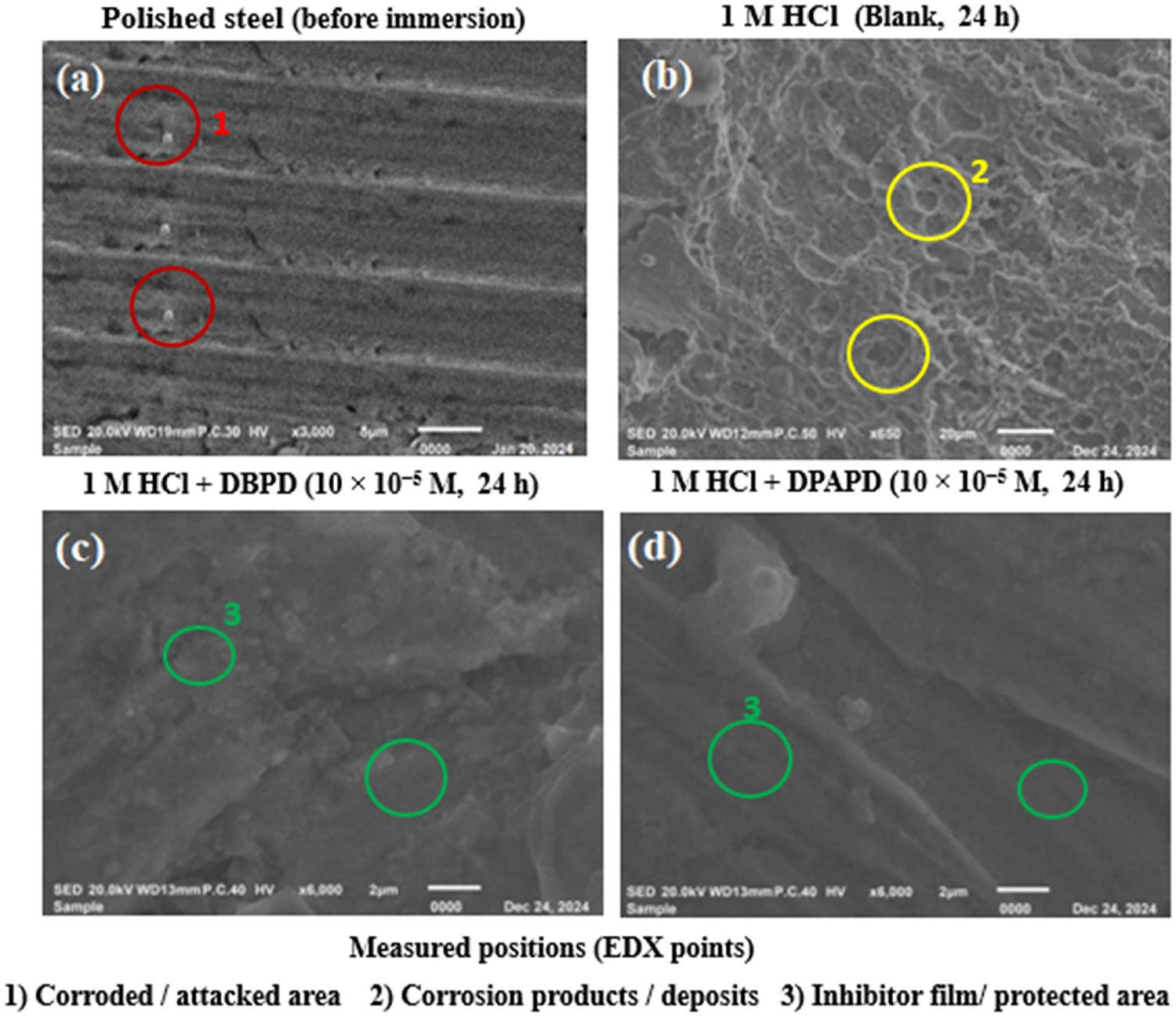
SEM micrographs of the carbon steel surface (**a**) before immersion in 1.0 M HCl, (**b**) after 24 h of immersion in 1.0 M HCl (c,d) after 24 h of immersion in 1.0 M HCl (10 × 10^−5^ M) of DBPD and DPAPD respectively, at 25 °C. The circles indicate the positions, where EDX analyses were performed.

The surface morphology of carbon steel specimens before and after exposure to the corrosive medium was examined using scanning electron microscopy (SEM) at a magnification of 500 × . The micrograph of the freshly polished steel surface (Fig. 11a) reveals a relatively smooth and homogeneous morphology, with only a few minor inclusions that may be attributed to residual oxide particles originating from the manufacturing process.

After immersion in 1.0 M HCl solution for 24 h in the absence of inhibitor, significant surface degradation is observed (Fig. 11b). The steel surface appears severely damaged, characterized by the presence of deep pits, irregular grooves, and a highly roughened texture. Dark regions are indicative of heavily corroded areas, while lighter patches can be associated with corrosion products such as iron oxides and hydroxides. This morphology clearly reflects the aggressive attack of the acidic medium on the unprotected metal surface. Additionally, exposure to air during the drying process may have promoted further oxidation, contributing to the formation of heterogeneous oxide layers and a flaky surface appearance.

In contrast, the SEM images of the specimens immersed in inhibited solutions containing higher concentrations (10 × 10^-5^ M) of DBPD and DPAPD (Fig. 11c and d, respectively) show a remarkable improvement in surface condition. The metal surfaces appear considerably smoother and more uniform, with a substantial reduction in corrosion-related defects. This observation indicates that both inhibitors effectively suppress acid-induced corrosion.

The enhanced surface integrity can be attributed to the adsorption of DBPD and DPAPD molecules onto the carbon steel surface, leading to the formation of a protective film that blocks active corrosion sites and restricts the diffusion of aggressive species such as H⁺ and Cl⁻ ions. The clear distinction between the uninhibited and inhibited samples provides strong visual confirmation of the high inhibition efficiency of the investigated bis-chalcone derivatives in acidic environments.

#### Energy dispersive X-ray spectroscopy

**Fig. 12:**
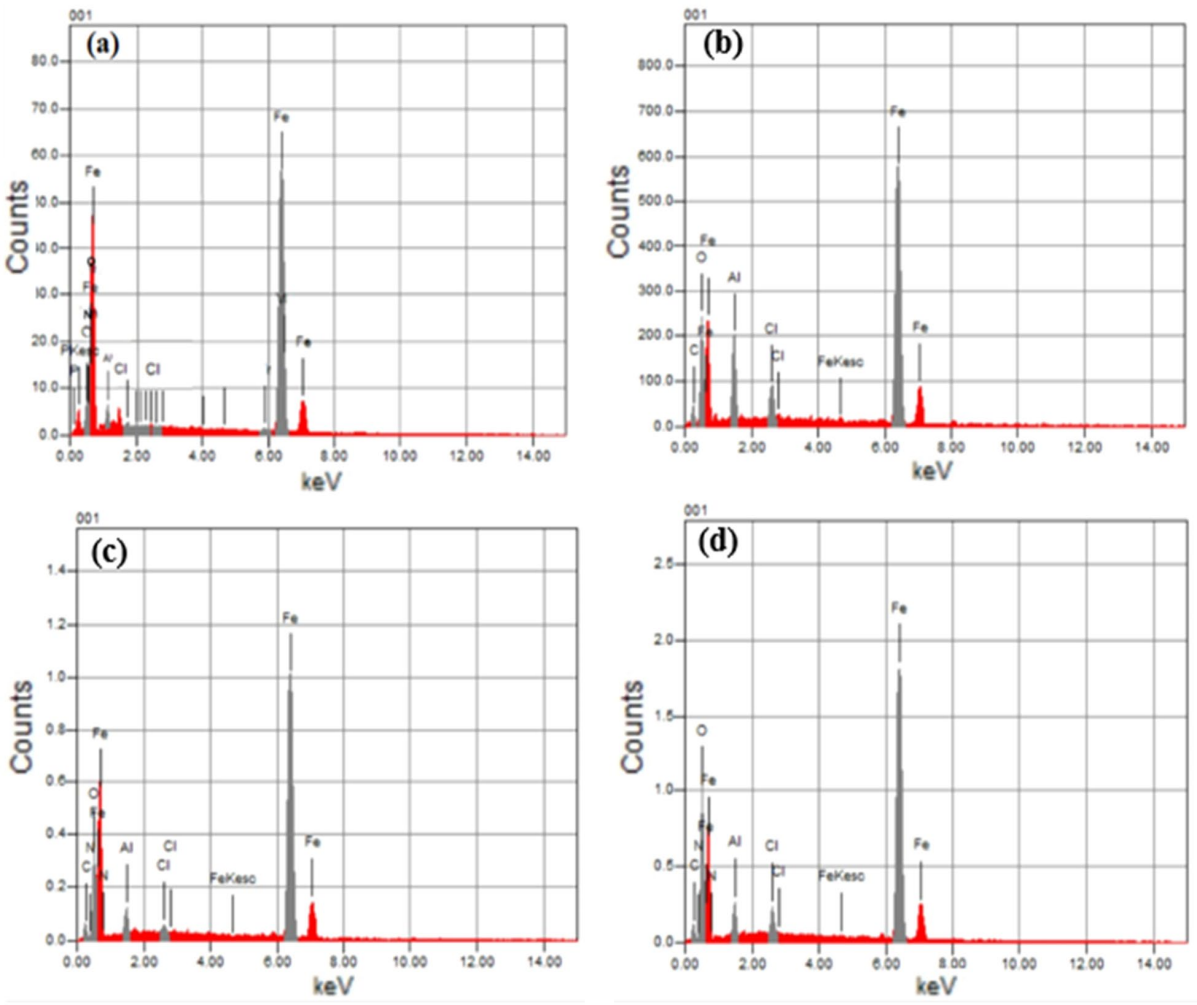
EDX analysis of the carbon steel surface (**a**) before immersion in 1.0 M HCl, (**b**) after 24 h of immersion in 1.0 M HCl, (**c**,**d**) after 24 h of immersion in 1.0 M HCl (10 × 10^−5^ M) of DBPD and DPAPD respectively, at 25 °C.

Energy-dispersive X-ray spectroscopy (EDX) was employed to analyze the elemental composition of carbon steel surfaces before and after immersion in 1.0 M HCl solution, in both the absence and presence of the investigated inhibitors. The corresponding EDX spectra are presented in Fig. 12a, b for the uninhibited system, and in Fig. 12c, d for samples exposed to inhibited solutions containing 10 × 10^-5^ M of DBPD and DPAPD, respectively.

The quantitative elemental data derived from the EDX measurements are summarized in Table 8. In the absence of inhibitors, a significant decrease in the iron content is observed, reaching 58.84%, which reflects severe surface degradation due to corrosion. This reduction is associated with the formation of corrosion products, such as iron oxides and chlorides, that partially cover the steel surface and attenuate the Fe signal detected during analysis.

**Table 8:** Percentage atomic contents of elements obtained from EDX spectra.

Inhibitor	Fe	Cl	Al	O	N	C
Carbon steel (free)	88.75	0.24	0.79	8.88	0.33	1.00
Carbon steel in 1.0 M HCl	58.84	0.61	8.77	17.36	1.55	12.87
DBPD	78.24	0.57	2.22	11.94	1.00	6.04
DPAPD	70.33	1.75	2.46	18.98	0.63	5.85

In contrast, the presence of DBPD and DPAPD leads to a marked increase in the detected iron content, rising to 78.24 and 70.33%, respectively. This enhancement indicates that the inhibitors effectively mitigate iron dissolution and suppress the formation of corrosion products, thereby preserving a higher fraction of the metallic surface. The increased Fe signal is indicative of reduced surface damage and confirms the protective role of the adsorbed inhibitor layer.

Additionally, the EDX spectra of the inhibited samples exhibit characteristic peaks corresponding to carbon (C), nitrogen (N), and oxygen (O), which can be attributed to the molecular structure of the inhibitor compounds. The presence of these elements on the steel surface provides clear evidence of successful adsorption and the formation of an adherent organic film. This film acts as a barrier that limits the interaction between the metal surface and the aggressive acidic environment, in agreement with the electrochemical and surface morphology results.

The EDX analysis strongly supports the proposed inhibition mechanism, demonstrating that DBPD and DPAPD protect carbon steel through adsorption and the formation of a stable and protective surface layer.

### Quantum chemical calculations

**Fig. 13:**
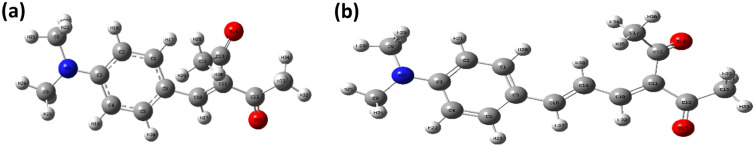
The final optimized structures of (**a**) DBPD and (**b**) DPAPD by B3LYP/6-311G**(d,p) level.

The corrosion inhibition of carbon steel employing the synthesized inhibitor was experimentally studied. Consequently, quantum chemical simulations were performed to explore the influence of structural factors on inhibitor effectiveness as well as the processes of adsorption on the metal surface. The geometric and electrical structures of the inhibitors were computed by optimizing their bond lengths and bond angles (Table S1 and S4, supplementary materials). The optimized molecule structures with the lowest energy derived from B3LYP/6-311G**(d,p) level (Fig. 13)^74^.

Table 9 gathers quantum chemical parameters derived from calculations that are responsible for inhibitor inhibition efficiency, such as the energies of the highest occupied molecular orbital (E_HOMO_), the energies of the lowest unoccupied molecular orbital (E_LUMO_), energy gap ΔE = (E_LUMO_ − E_HOMO_) which represents the function of reactivity, electronegativity (χ), dipole moment (D), softness (σ), chemical potential (μ), and hardness (η). According to Koopman’s theorem^75^, the inhibitor molecule’s E_HOMO_ and E_LUMO_ are connected to its the electron affinity (A), and ionisation potential (I), respectively. Further quantum chemical characteristics that give important information about the reactive activity of the inhibitors and were determined using the following relationships^76,77^:

$$\upchi (\mathrm{e}\mathrm{l}\mathrm{e}\mathrm{c}\mathrm{t}\mathrm{r}\mathrm{o}\mathrm{n}\mathrm{e}\mathrm{g}\mathrm{a}\mathrm{t}\mathrm{i}\mathrm{v}\mathrm{i}\mathrm{t}\mathrm{y}) = \frac{-({E}_{LUMO} + {E}_{HOMO})}{2}$$

$$\upmu (\mathrm{p}\mathrm{o}\mathrm{t}\mathrm{e}\mathrm{n}\mathrm{t}\mathrm{i}\mathrm{a}\mathrm{l}) = - \upchi = \frac{\left({E}_{LUMO} + {E}_{HOMO}\right)}{2}$$

$$\upeta (\mathrm{h}\mathrm{a}\mathrm{r}\mathrm{d}\mathrm{n}\mathrm{e}\mathrm{s}\mathrm{s}) = \frac{({E}_{LUMO}- {E}_{HOMO})}{2}$$

$$\omega \left( {{\mathrm{electrophilicity}}} \right) = \mu^{{2}} /{2}\eta$$

**Table 9:** The calculated quantum chemical parameters obtained from B3LYP/6-311G**(d,p) calculations for DBPD and DPAPD.

Inhibitor	E_HOMO_ (eV)	E_LUMO_ (eV)	ΔE (eV)	D (Debye)	η (eV)	σ (eV^-1^)	μ (eV)	χ (eV)	ω (eV)	∆*N*
DBPD	− 5.710	− 2.099	3.611	7.47895	1.806	0.554	− 3.904	3.904	4.221	0.254
DPAPD	− 6.881	− 2.498	4.383	10.1756	2.192	0.456	− 4.689	4.689	5.017	0.030

The softness is defined as the inverse of the global hardness, which is as follows:

$$\sigma \left( {{\mathrm{softness}}} \right)\, = \,{1}/\eta$$

The fraction of electrons (Δ*N*) exchanged between the inhibitor and the metallic surface is calculated as^78,79^:

$$\Delta N=\frac{({\upchi}_{Fe}-{\upchi}_{inh})}{2({\upeta}_{Fe}+{\upeta}_{inh})}= \frac{(\Phi -{\upchi}_{inh})}{2{\upeta}_{inh}}$$

where a theoretical value of $\Phi$=4.82eV, and η_Fe_ = 0 is taken based on the assumption that I = A for a bulk metal, because they are softer than the neutral metallic atoms.

Chemical reactivity, according to the frontier molecular orbital theory, is a result of the interaction between the HOMO and LUMO levels of the reacting species^80^. With empty molecular orbitals, E_HOMO_ denotes the molecule’s capacity to give electrons to a suitable acceptor, and E_LUMO_ denotes its ability to take electrons. The stronger the molecule’s capacity to receive electrons, the lower the value of E_LUMO_^81^. The higher the inhibitor’s E_HOMO_ , the easier it is to give electrons to the metal surface’s empty d-orbital and the better its inhibition effectiveness.

Table 9 reveals that the studied inhibitor possesses the E_HOMO_ (-5.710 and -6.881 eV) for DBPD and DPAPD, respectively. This explains its inclination to adsorb on metal surfaces, as well as its inhibitory efficiency. The ΔE gap = E_HOMO_–E_LUMO_ is a crucial stability measure used to create theoretical models for describing the structure and conformational barriers in many molecular systems. The lower the value of ΔE , the more likely the molecule is to be effective at inhibiting^82,83^. Calculations indicated that the inhibitors; DBPD and DPAPD have an energy gap (3.611 and 4.383 eV, respectively) As a result, the inhibitors are predicted to preferentially bind to the metal surface.

Absolute hardness, η, and softness, σ, are crucial qualities for determining the stability and reactivity of a molecule. The energy gap in a hard molecule is large, whereas the energy gap in a soft molecule is small. Because they may easily supply electrons to an acceptor, soft molecules are more reactive than hard molecules Adsorption occurs in the portion of the molecule with the largest value of σ, which is a local characteristic^81^. The metal functions as a Lewis acid in a corrosion system, whereas the inhibitor works as a Lewis base. Because bulk metals are soft acids, soft base inhibitors are particularly effective against acidic corrosion of those metals. Therefore, the σ values of DBPD and DPAPD (0.554 and 0.456 eV^−1^), respectively show the inhibitor’s inhibitory effectiveness. Additionally, the calculations found that the inhibitors possesses χ (3.904 and 4.689 eV) for DBPD and DPAPD, respectively, which explains the inhibitor’s capacity to donate to the metal surface.

If Δ*N* > 0, the electron transfer from inhibitor to metal is measured, and vice versa if Δ*N* < 0. Furthermore, Lukovits, et al^84^ find that when ΔN < 3.6, the inhibition effectiveness improves as the ability of electron donation to the metal surface increases. Table 9 shows that all the Δ*N* values for both inhibitors are positive, indicating that the inhibitor can donate electrons to the iron surface to form self-assembled inhibitor layers. However, the Δ*N* value of the inhibitor is lower than 3.6, indicating that the inhibitor’s inhibition efficiency increases with the ability of electron donation to the metal surface.

**Fig. 14:**
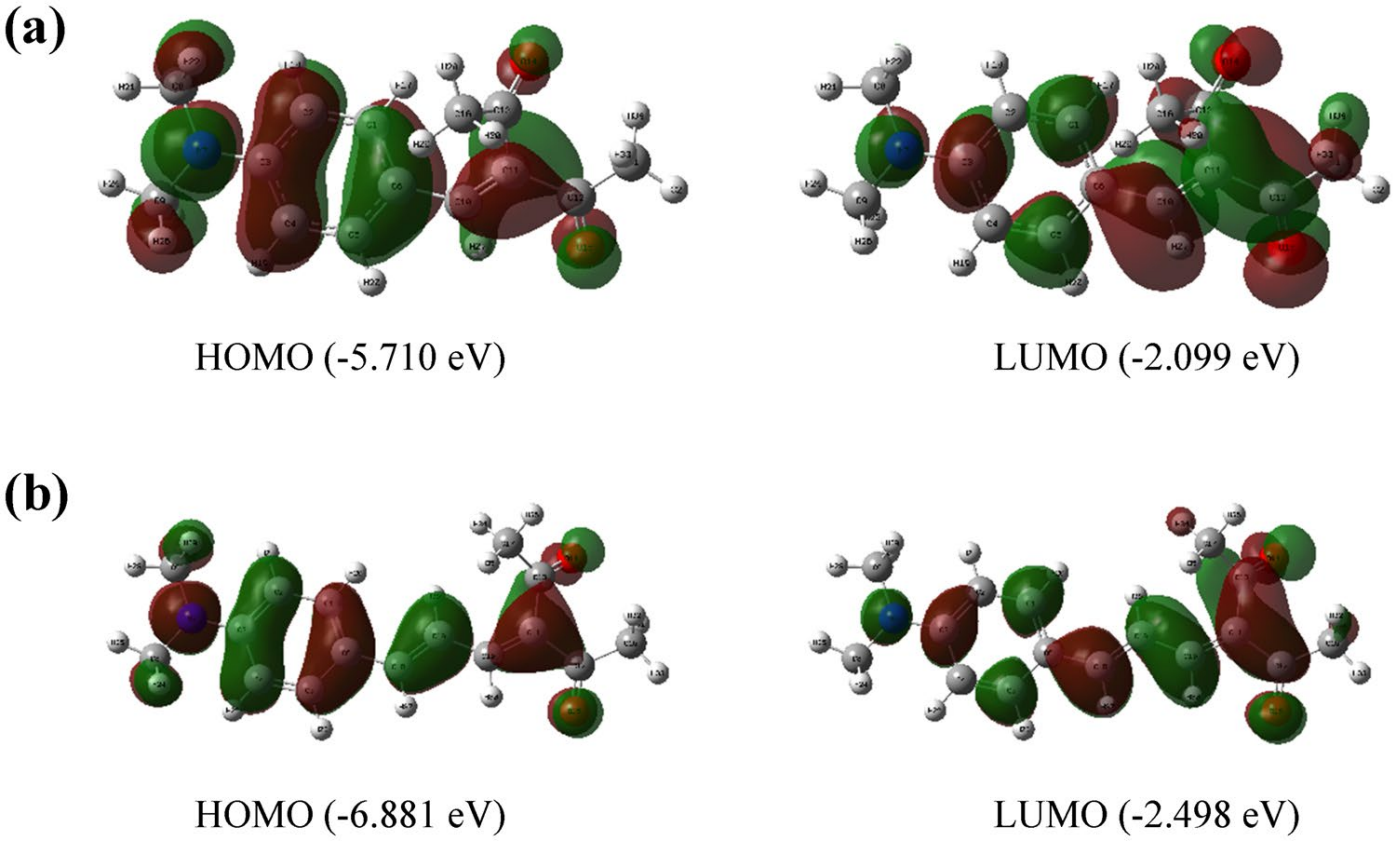
3D plots frontier orbital energies of (**a**) DBPD and (**b**) DPAPD by B3LYP/6-311G^**^(d,p) level.

Furthermore, as shown in Fig. 14, the HOMO level of all inhibitors is largely localised on the central aromatic ring, indicating that this ring is the favored location for electrophilic assault at the metal surface. This suggests that aromatic rings with high HOMO density coefficients are orientated toward the metal surface, and adsorption most likely happened through the aromatic ring’s π-electrons. Additionally, the calculations indicated that the LUMO level’s charge density is totally delocalized from the central aromatic ring, hinting that this moiety might respond as an electrophile (electron acceptor).

**Fig. 15:**
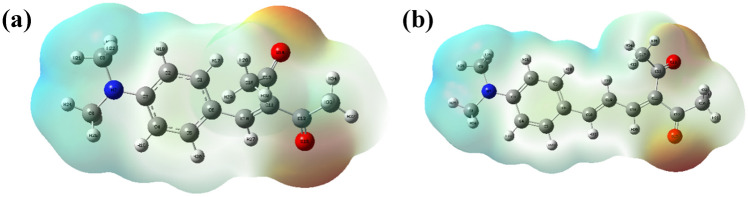
Molecular electrostatic potential of DBPD and DPAPD by B3LYP/6-311G^**^(d,p) level.

The molecular electrostatic potentials (MEPs) (Fig. 15) are extremely important because negative electrostatic potential areas may be thought of as nucleophilic centers, whilst positive electrostatic potential regions can be thought of as potential electrophilic sites. Furthermore, the electrostatic potential displays the electron density’s polarization.

In descending sequence, the electrical density is as follows: red > orange > yellow > green > blue. The negative sections (in red) are electrophilic, whereas the positive regions (in blue) are electron poor. According to the study of this picture, the high electron density (red color) is delocalized in the electron rich areas with oxygen atoms of both inhibitors and their surrounding atoms. Low electron density distinguishes the remaining places. These high-density electronic atoms will form an electronic connection with the iron’s unoccupied d-orbitals, implying that adsorption between the inhibitor molecule and the iron surface happens spontaneously^85^.

### Molecular dynamic simulations

**Fig. 16:**
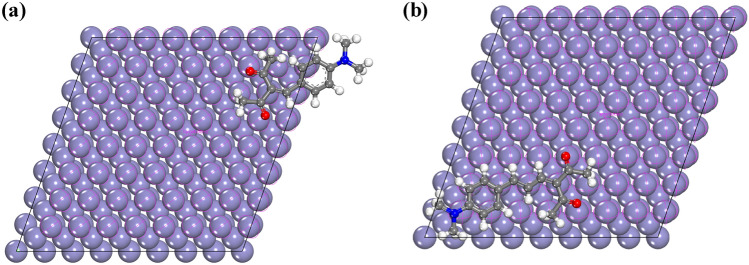
Molecular simulations for the most favorable modes of adsorption obtained for the inhibitor on Fe (110) surface, (**a**) DBPD, and (**b**) DPAPD.

The inhibitors investigated have a variety of active locations for adsorption on the metal surface. Consequently, molecular dynamics were run on a system comprising the examined inhibitor and the iron surface to determine the optimal adsorption site for the inhibitor compound’s interaction with the Fe(110) surface. The adsorbate components’ structures were decreased until they fulfilled specified criteria. Figure 16 depicts the top views of the inhibitors; DBPD and DPAPD adsorption modeling on the surface of Fe (110). The inhibitor can be adsorbed on the iron surface via the charge of the central aromatic ring and the lone pairs of oxygen atoms, according to this picture. These interactions can result in the keto-complex establishing coordinated bonds with the iron surface and generating a tight layer that prevents corrosive media from moving to the metal surface.

The following relationship was used to compute the interaction energy (i.e., binding energy) between the inhibitor molecule and the iron surface:

$${\mathrm{E}}_{{{\mathrm{ads}}}} = {\mathrm{E}}_{{{\text{Fe }}-{\mathrm{inh}}}} - \left( {{\mathrm{E}}_{{{\mathrm{inh}}}} + {\mathrm{E}}_{{{\mathrm{Fe}}}} } \right)$$

where E_inh_ and E_Fe_ are the total energies of the inhibitor and Fe surface, respectively. It was possible to get a very broad contact energy (i.e., binding energy). The (E_bind_ =  − E_ads_), which is the negative value of adsorption energy^86^, explains the spontaneity of the inhibitor molecule adsorption on the metal surface as well as the extremely high inhibition effectiveness^87^.

**Fig. 17:**
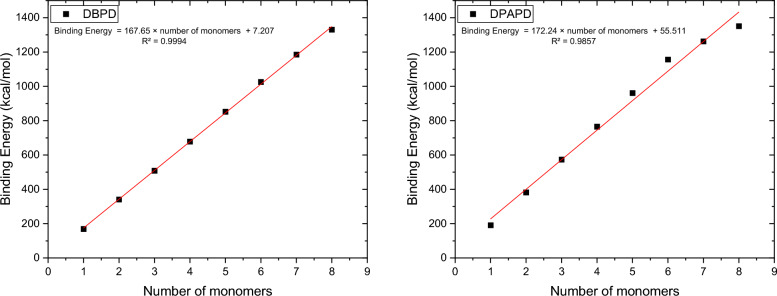
The binding energies according to the number of monomers of the inhibitors (DBPD and DPAPD) on the Fe(110) surface.

Tables 10 and 11 illustrate the binding and adsorption energies obtained by increasing the number of inhibitor monomers up to eight, while increasing the number beyond eight resulted in the failure of the MD interaction run. The adsorption energy increases with increasing monomer concentration, which is consistent with the experimental results. This behavior can be attributed to the increase in the number of functional groups available for interaction, indicating the stability and spontaneity of the formed complex. Figure 17 shows that the MD results correlate well with the experimental data.

**Table 10:** Outputs and descriptors calculated by the Monte Carlo simulation for adsorption of the inhibitor (DBPD) on Fe (110) surface with increasing if inhibitor monomer numbers.

# Inhibitor monomers	Total energy (Kcal/mol)	Adsorption energy (Kcal/mol)	Binding energy (Kcal/mol)	Rigid adsorption energy (Kcal/mol)	Deformation energy (Kcal/mol)	inhibitor: dE_ad_/dNi
1	− 233.805	− 169.316	169.316	− 146.128	− 23.188	− 169.316
2	− 469.843	− 340.865	340.865	− 296.089	− 44.776	− 171.724
3	− 702.256	− 508.790	508.790	− 442.149	− 66.641	− 168.319
4	− 936.591	− 678.636	678.636	− 589.301	− 89.334	− 168.395
5	− 1175.172	− 852.728	852.728	− 740.049	− 112.679	− 172.210
6	− 1412.889	− 1025.956	1025.956	− 893.388	− 132.569	− 173.194
7	− 1637.352	− 1185.930	1185.930	− 1021.898	− 164.032	− 168.681
8	− 1846.971	− 1331.061	1331.061	− 1130.623	− 200.437	− 159.023

**Table 11:** Outputs and descriptors calculated by the Monte Carlo simulation for adsorption of the inhibitor (DPAPD) on Fe (110) surface with increasing if inhibitor monomer numbers.

# Inhibitor monomers	Total energy (Kcal/mol)	Adsorption energy (Kcal/mol)	Binding energy (Kcal/mol)	Rigid adsorption energy (Kcal/mol)	Deformation energy (Kcal/mol)	inhibitor: dE_ad_/dNi
1	− 261.379	− 191.282	191.282	− 168.997	− 22.285	− 191.282
2	− 522.217	− 382.021	382.021	− 337.263	− 44.758	− 191.039
3	− 784.103	− 573.810	573.810	− 507.433	− 66.377	− 191.291
4	− 1046.013	− 765.622	765.622	− 676.426	− 89.196	− 193.015
5	− 1312.001	− 961.512	961.512	− 848.914	− 112.598	− 192.092
6	− 1577.239	− 1156.653	1156.653	− 1024.181	− 132.472	− 194.006
7	− 1753.276	− 1262.592	1262.592	− 1116.259	− 146.333	− 128.991
8	− 1912.275	− 1351.494	1351.494	− 1183.468	− 168.026	− 93.401

**Fig. 18:**
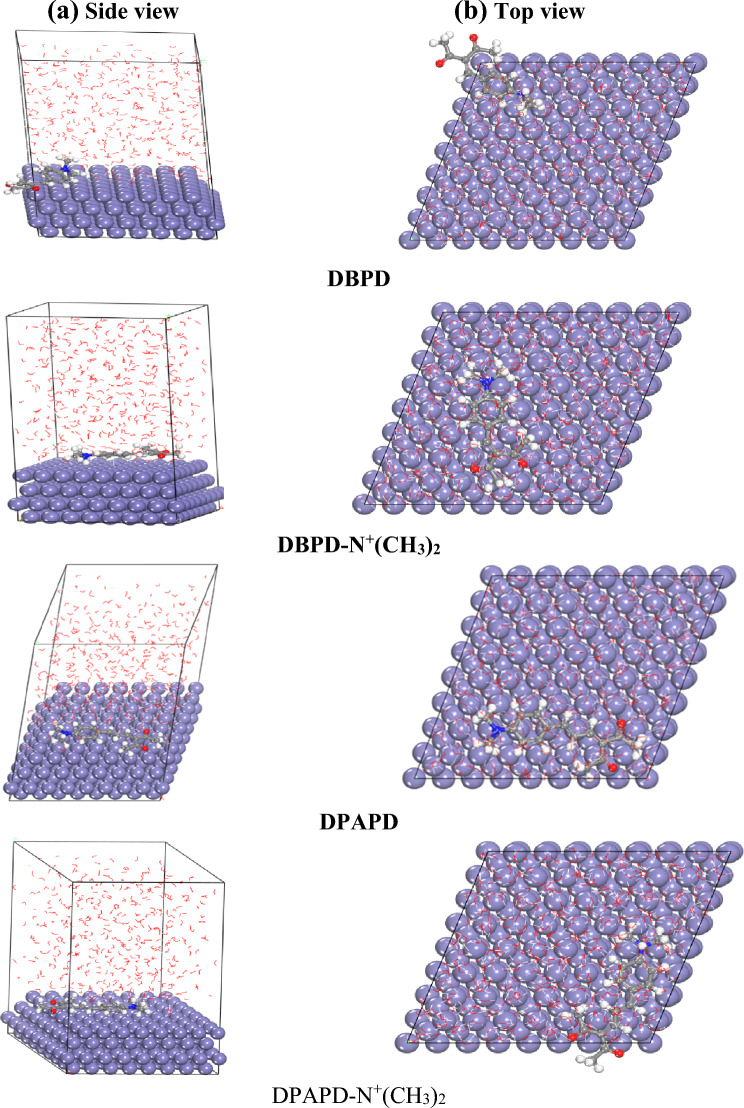
Molecular simulations for the most favorable modes of adsorption obtained for the inhibitors; DBPD, DBPD-N^+^(CH_3_)_2_, DPAPD and DPAPD-N^+^(CH_3_)_2_ on Fe (110) surface, side view (**a**) and top view (**b**).

Considering that the corrosion process occurs in an acidic medium, additional Monte Carlo simulations were performed for the protonated forms of the investigated inhibitors (DBPD-N^+^(CH_3_)_2_ and DPAPD-N^+^(CH_3_)_2_). The corresponding adsorption parameters for both neutral and protonated species are presented in Table 12, while the optimized adsorption configurations are shown in Fig. 18.

**Table 12:** Outputs and descriptors calculated by the Monte Carlo simulation for adsorption of the inhibitor (DBPD, DBPD-N^+^(CH_3_)_2_, DPAPD and DPAPD-N^+^(CH_3_)_2_ on Fe (110) surface.

compound	Total energy (Kcal/mol)	Adsorption energy (Kcal/mol)	Binging energy (Kcal/mol)	Rigid adsorption energy (Kcal/mol)	Deformation energy (Kcal/mol)	Inhibitor: dEad/dNi	H_3_O^+^: dEad/dNi	HCl: dEad/dNi	H_2_O: dEad/dNi
DBPD	− 1240.271	− 1462.573	1462.573	− 1159.983	− 302.590	− 178.764	− 121.125	− 14.427	− 26.722
DBPD-N^+^(CH_3_)_2_	− 1224.901	− 1447.204	1447.204	− 1155.497	− 291.707	− 182.844	− 91.706	− 13.705	− 22.171
DPAPD	− 1257.765	− 1474.459	1474.459	− 1184.963	− 289.496	− 209.806	− 93.871	− 14.155	− 30.595
DPAPD-N^+^(CH_3_)_2_	− 1267.813	− 1484.506	1484.506	− 1192.357	− 292.149	− 215.152	− 120.763	− 14.275	− 30.802

The higher the binding energy value, the stronger the adsorption of the inhibitor on the metal surface and consequently the higher the inhibition efficiency. For the neutral species, DPAPD exhibits a slightly higher binding energy than DBPD, in good agreement with the experimental inhibition efficiencies. The inclusion of protonated species further confirms the adsorption tendency under acidic conditions. The calculated binding energies follow the overall order:

$${\mathrm{DPAPD}} - {\mathrm{N}}^{ + } \left( {{\mathrm{CH}}_{{3}} } \right)_{{2}} > {\mathrm{DPAPD}} > {\mathrm{DBPD}} > {\mathrm{DBPD}} - {\mathrm{N}}^{ + } \left( {{\mathrm{CH}}_{{3}} } \right)_{{2}}$$

This trend is consistent with the experimental results and highlights DPAPD as the most strongly adsorbed inhibitor on the iron surface. Figure 18 presents the most favorable adsorption configurations of both neutral and protonated inhibitor species on the Fe(110) surface. From the top-view images, DPAPD is seen to cover a larger surface area of Fe(110) than DBPD, leading to the formation of a more compact protective layer and thus higher corrosion inhibition efficiency.

## Conclusion

In this work, two bis-chalcone derivatives, DBPD and DPAPD, were systematically investigated as corrosion inhibitors for carbon steel in 1.0 M HCl solution using combined experimental and theoretical approaches. Both compounds exhibited excellent inhibition performance, with DPAPD showing superior efficiency exceeding 90% at 298 K. Electrochemical measurements demonstrated that the inhibitors act as mixed-type inhibitors and protect the steel surface through adsorption-driven protective film formation. Adsorption followed the Langmuir isotherm model and proceeded spontaneously with exothermic behavior. Surface analyses confirmed the formation of a compact and adherent protective layer that effectively reduced corrosion damage.

Theoretical calculations, including quantum chemical parameters and molecular simulations, further supported the experimental findings by revealing strong interactions between the inhibitor molecules and the Fe(110) surface. In particular, molecular dynamics and Monte Carlo simulations indicated that both neutral and protonated species contribute to the adsorption process, with enhanced interaction strength observed in acidic conditions, especially for DPAPD. The close agreement between experimental and computational results provides a comprehensive understanding of the inhibition mechanism and highlights the influence of molecular structure as well as protonation state on inhibition performance.

These findings demonstrate the potential of bis-chalcone derivatives as efficient and structurally tunable corrosion inhibitors for acidic industrial environments and provide useful guidelines for the rational design of next-generation organic corrosion inhibitors.

## Supplementary Information

Supplementary Information.

## Data Availability

All data generated or analyzed during this study are included within the manuscript and its supplementary materials.
