# Supplementary material for: Synergistic experimental and theoretical investigation of bis-chalcone derivatives as efficient corrosion inhibitors for carbon steel in acidic media
Source: Sci Rep. 2026 Jul 18;16:22543. doi: 10.1038/s41598-026-60964-2 (PMC13380626; doi:10.1038/s41598-026-60964-2)
Supplement: Supplementary file 1 — Supplementary Information. [file 41598_2026_60964_MOESM1_ESM.docx]

**Supplementary Materials**

Table S1. Optimized bond lengths (Å) of DBPD.

| **Bond** | **Length(Å)** | **Bond** | **Length(Å)** | **Bond** | **Length(Å)** |
| --- | --- | --- | --- | --- | --- |
| C(31)-H(34) | 1.092 | C(31)-H(33) | 1.095 | C(31)-H(32) | 1.089 |
| C(16)-H(30) | 1.097 | C(16)-H(29) | 1.09 | C(16)-H(28) | 1.09 |
| C(13)-C(16) | 1.518 | C(13)-O(14) | 1.217 | C(12)-C(31) | 1.514 |
| C(12)-O(15) | 1.219 | C(11)-C(13) | 1.502 | C(11)-C(12) | 1.508 |
| C(10)-H(27) | 1.089 | C(10)-C(11) | 1.357 | C(9)-H(26) | 1.094 |
| C(9)-H(25) | 1.098 | C(9)-H(24) | 1.089 | C(8)-H(23) | 1.098 |
| C(8)-H(22) | 1.094 | C(8)-H(21) | 1.089 | N(7)-C(9) | 1.456 |
| N(7)-C(8) | 1.456 | C(6)-C(10) | 1.451 | C(5)-H(20) | 1.085 |
| C(5)-C(6) | 1.409 | C(4)-H(19) | 1.081 | C(4)-C(5) | 1.383 |
| C(3)-N(7) | 1.377 | C(3)-C(4) | 1.416 | C(2)-H(18) | 1.081 |
| C(2)-C(3) | 1.417 | C(1)-H(17) | 1.083 | C(1)-C(6) | 1.409 |
| C(1)-C(2) | 1.383 |  |  |  |  |

Table S2. Optimized bond angles (**°**) of DBPD

| **Angle** | **Degree (°)** | **Angle** | **Degree (°)** | **Angle** | **Degree (°)** |
| --- | --- | --- | --- | --- | --- |
| H(34)-C(31)-H(33) | 106.625 | H(34)-C(31)-H(32) | 110.385 | H(34)-C(31)-C(12) | 110.462 |
| H(33)-C(31)-H(32) | 109.627 | H(33)-C(31)-C(12) | 110.931 | H(32)-C(31)-C(12) | 108.8 |
| H(30)-C(16)-H(29) | 107.214 | H(30)-C(16)-H(28) | 108.534 | H(30)-C(16)-C(13) | 107.69 |
| H(29)-C(16)-H(28) | 110.801 | H(29)-C(16)-C(13) | 112.334 | H(28)-C(16)-C(13) | 110.105 |
| C(16)-C(13)-O(14) | 121.119 | C(16)-C(13)-C(11) | 117.156 | O(14)-C(13)-C(11) | 121.543 |
| C(31)-C(12)-O(15) | 120.549 | C(31)-C(12)-C(11) | 118.55 | O(15)-C(12)-C(11) | 120.896 |
| C(13)-C(11)-C(12) | 118.761 | C(13)-C(11)-C(10) | 124.26 | C(12)-C(11)-C(10) | 116.979 |
| H(27)-C(10)-C(11) | 113.986 | H(27)-C(10)-C(6) | 115.226 | C(11)-C(10)-C(6) | 130.768 |
| H(26)-C(9)-H(25) | 108.269 | H(26)-C(9)-H(24) | 107.625 | H(26)-C(9)-N(7) | 111.151 |
| H(25)-C(9)-H(24) | 108.249 | H(25)-C(9)-N(7) | 112.367 | H(24)-C(9)-N(7) | 109.029 |
| H(23)-C(8)-H(22) | 108.275 | H(23)-C(8)-H(21) | 108.214 | H(23)-C(8)-N(7) | 112.468 |
| H(22)-C(8)-H(21) | 107.626 | H(22)-C(8)-N(7) | 111.073 | H(21)-C(8)-N(7) | 109.03 |
| C(9)-N(7)-C(8) | 118.492 | C(9)-N(7)-C(3) | 120.006 | C(8)-N(7)-C(3) | 120.077 |
| C(10)-C(6)-C(5) | 118.721 | C(10)-C(6)-C(1) | 124.696 | C(5)-C(6)-C(1) | 116.537 |
| H(20)-C(5)-C(6) | 118.88 | H(20)-C(5)-C(4) | 118.798 | C(6)-C(5)-C(4) | 122.319 |
| H(19)-C(4)-C(5) | 118.711 | H(19)-C(4)-C(3) | 120.427 | C(5)-C(4)-C(3) | 120.858 |
| N(7)-C(3)-C(4) | 121.487 | N(7)-C(3)-C(2) | 121.465 | C(4)-C(3)-C(2) | 117.045 |
| H(18)-C(2)-C(3) | 120.3 | H(18)-C(2)-C(1) | 118.441 | C(3)-C(2)-C(1) | 121.246 |
| H(17)-C(1)-C(6) | 119.95 | H(17)-C(1)-C(2) | 118.084 | C(6)-C(1)-C(2) | 121.902 |

Table S3. Optimized bond lengths (Å) of DPAPD.

| **Bond** | **Length(Å)** | **Bond** | **Length(Å)** | **Bond** | **Length(Å)** |
| --- | --- | --- | --- | --- | --- |
| C(19)-H(38) | 1.083 | C(18)-H(37) | 1.089 | C(18)-C(19) | 1.361 |
| C(17)-H(36) | 1.092 | C(17)-H(35) | 1.09 | C(17)-H(34) | 1.095 |
| C(16)-H(33) | 1.089 | C(16)-H(32) | 1.093 | C(16)-H(31) | 1.092 |
| C(13)-C(17) | 1.524 | C(13)-O(14) | 1.22 | C(12)-C(16) | 1.514 |
| C(12)-O(15) | 1.221 | C(11)-C(13) | 1.491 | C(11)-C(12) | 1.509 |
| C(10)-H(30) | 1.089 | C(10)-C(19) | 1.43 | C(10)-C(11) | 1.366 |
| C(9)-H(29) | 1.094 | C(9)-H(28) | 1.089 | C(9)-H(27) | 1.098 |
| C(8)-H(26) | 1.098 | C(8)-H(25) | 1.089 | C(8)-H(24) | 1.094 |
| N(7)-C(9) | 1.456 | N(7)-C(8) | 1.455 | C(6)-C(18) | 1.447 |
| C(5)-H(23) | 1.085 | C(5)-C(6) | 1.408 | C(4)-H(22) | 1.081 |
| C(4)-C(5) | 1.383 | C(3)-N(7) | 1.376 | C(3)-C(4) | 1.415 |
| C(2)-H(21) | 1.081 | C(2)-C(3) | 1.419 | C(1)-H(20) | 1.084 |
| C(1)-C(6) | 1.41 | C(1)-C(2) | 1.381 |  |  |

Table S4. Optimized bond angles (**°**) of DPAPD

| **Angle** | **Degree (°)** | **Angle** | **Degree (°)** | **Angle** | **Degree (°)** |
| --- | --- | --- | --- | --- | --- |
| H(38)-C(19)-C(18) | 119.504 | H(38)-C(19)-C(10) | 119.497 | C(18)-C(19)-C(10) | 120.99 |
| H(37)-C(18)-C(19) | 116.833 | H(37)-C(18)-C(6) | 114.961 | C(19)-C(18)-C(6) | 128.206 |
| H(36)-C(17)-H(35) | 109.836 | H(36)-C(17)-H(34) | 106.804 | H(36)-C(17)-C(13) | 107.623 |
| H(35)-C(17)-H(34) | 109.239 | H(35)-C(17)-C(13) | 113.614 | H(34)-C(17)-C(13) | 109.496 |
| H(33)-C(16)-H(32) | 110.177 | H(33)-C(16)-H(31) | 110.186 | H(33)-C(16)-C(12) | 108.276 |
| H(32)-C(16)-H(31) | 106.411 | H(32)-C(16)-C(12) | 111.572 | H(31)-C(16)-C(12) | 110.223 |
| C(17)-C(13)-O(14) | 118.952 | C(17)-C(13)-C(11) | 120.016 | O(14)-C(13)-C(11) | 121.006 |
| C(16)-C(12)-O(15) | 119.877 | C(16)-C(12)-C(11) | 119.833 | O(15)-C(12)-C(11) | 120.259 |
| C(13)-C(11)-C(12) | 119.874 | C(13)-C(11)-C(10) | 124.077 | C(12)-C(11)-C(10) | 115.977 |
| H(30)-C(10)-C(19) | 115.669 | H(30)-C(10)-C(11) | 113.973 | C(19)-C(10)-C(11) | 130.288 |
| H(29)-C(9)-H(28) | 107.654 | H(29)-C(9)-H(27) | 108.305 | H(29)-C(9)-N(7) | 111.281 |
| H(28)-C(9)-H(27) | 108.178 | H(28)-C(9)-N(7) | 109.037 | H(27)-C(9)-N(7) | 112.236 |
| H(26)-C(8)-H(25) | 108.204 | H(26)-C(8)-H(24) | 108.253 | H(26)-C(8)-N(7) | 112.266 |
| H(25)-C(8)-H(24) | 107.681 | H(25)-C(8)-N(7) | 109.075 | H(24)-C(8)-N(7) | 111.216 |
| C(9)-N(7)-C(8) | 118.782 | C(9)-N(7)-C(3) | 120.207 | C(8)-N(7)-C(3) | 120.095 |
| C(18)-C(6)-C(5) | 119.488 | C(18)-C(6)-C(1) | 123.981 | C(5)-C(6)-C(1) | 116.53 |
| H(23)-C(5)-C(6) | 118.926 | H(23)-C(5)-C(4) | 118.699 | C(6)-C(5)-C(4) | 122.375 |
| H(22)-C(4)-C(5) | 118.728 | H(22)-C(4)-C(3) | 120.44 | C(5)-C(4)-C(3) | 120.832 |
| N(7)-C(3)-C(4) | 121.582 | N(7)-C(3)-C(2) | 121.352 | C(4)-C(3)-C(2) | 117.066 |
| H(21)-C(2)-C(3) | 120.209 | H(21)-C(2)-C(1) | 118.569 | C(3)-C(2)-C(1) | 121.222 |
| H(20)-C(1)-C(6) | 119.9 | H(20)-C(1)-C(2) | 118.13 | C(6)-C(1)-C(2) | 121.97 |
